# Investigating the Potential of Curcumin in the Treatment of Nonsmall Cell Lung Cancer: A Systematic Review With Meta‐Analysis, Network Pharmacology, and Mendelian Randomization

**DOI:** 10.1002/ptr.70073

**Published:** 2025-09-09

**Authors:** Yonglu Guo, Haohao Xu, Peng Shen, Ruijun Cai

**Affiliations:** ^1^ Department of Respiratory and Crical Care Medicine Shanghai General Hospital Jiuquan Hospital (The People's Hospital of Jiuquan) Jiuquan China; ^2^ College of Medical Technology, Qiqihar Medical University Qiqihar China; ^3^ The First Psychiatric Hospital of Harbin Harbin China; ^4^ Department of Pharmacy Shanghai General Hospital Jiuquan Hospital (The People's Hospital of Jiuquan) Jiuquan China

**Keywords:** curcumin, mendelian randomization, meta‐analysis, network pharmacology, nonsmall cell lung cancer (NSCLC), systematic review

## Abstract

To evaluate the efficacy and explore the potential mechanism of curcumin for the treatment and prevention of NSCLC. We searched six databases thoroughly for articles published before December 2024. Stata 15.0 software was applied for systematic review with meta‐analysis. We utilized network pharmacology, Mendelian randomization, and molecular docking techniques to investigate the pharmacological properties and potential targets of curcumin in the treatment of NSCLC. Twenty‐four studies involving 392 experimental animals were included in this study. Meta‐analysis results showed that curcumin significantly reduced tumor volume of mice (*p* < 0.001) in the NSCLC group compared to the control group. Two hundred twenty‐nine curcumin target genes were predicted. 1546 NSCLC‐related genes were obtained by taking the intersection of DEGs and genes in the key module of WGCNA. Eight hub genes were identified by protein–protein interaction. The eight hub genes showed significant clinical value and were found to be negatively correlated with the majority of immune cell infiltration. Molecular docking results indicated a good binding affinity between these eight hub genes and curcumin. Mendelian randomization demonstrated a causal relationship between the AURKB level and the increased risk of NSCLC. In this study, the effectiveness of curcumin has been demonstrated in in vivo models of NSCLC by meta‐analysis. AURKB has been identified as a high‐risk target for NSCLC by network pharmacological analysis and MR for the first time. Our study provides a scientific basis for clinical applications of curcumin in NSCLC.

## 1 Introduction

Lung cancer is both the most common and infamously difficult kind of respiratory cancer to cure. Most recent WHO statistics show that lung cancer is one of the cancer killers worldwide. Regrettably, the prognosis for this condition is rather bleak, with only a 10%–20% chance of surviving within 5 years of diagnosis in the majority of countries (Sung et al. 2021). NSCLC represents more than 85% of all reported instances of lung cancer (Schabath and Cote 2019). The main treatment options include mitomycin, gemcitabine, isophosphamide, and cisplatin. Alternatively, secondary or tertiary therapy options may include targeted drugs such as EGFR inhibitors, vascular endothelial growth factor (VEGF) receptor inhibitors, and ALK inhibitors. Immunotherapy, surgery, and traditional Chinese medicine (TCM) treatment are also considered additional treatment options (Goldstraw et al. 2016; Pignon et al. 2008). The occurrences of multidrug‐resistant and cross‐resistant NSCLC are gradually rising, which necessitates more effective treatment protocols in NSCLC of complex etiology and pathophysiology. The present focus of lung cancer research is the development of novel, highly sensitive treatments with few side effects.

Curcumin is a phenolic antioxidant that occurs naturally in the *Curcuma longa* , mustard, curry, and tulip plants from the Zingiberaceae plants family. It possesses distinct biological and medicinal properties (Aggarwal et al. 2007; Itokawa et al. 2008). Curcumin exhibits a range of pharmacological effects, including anti‐inflammatory, immune regulation, antioxidant, lipid‐modifying, and antitumor effects (Ganjali et al. 2014; Sahebkar 2015; Tu et al. 2019; Vadukoot et al. 2022; Zhang et al. 2016). Curcumin has diverse mechanisms and action channels to prevent and cure NSCLC. This preclinical study confirmed the retardation of NSCLC cell growth, spreading, curcumin‐induced programmed cell death, and exerted control over autophagy (Tang et al. 2022). Additionally, curcumin can impede the formation of tumor nodules (Chen et al. 2020), induce the production of reactive oxygen species and endoplasmic reticulum stress (Wu et al. 2010), regulate cell cycle progression (Liu et al. 2012), inhibit the migration of cancer cells (Chen et al. 2019), initiate apoptosis (Ye et al. 2015), and enhance DNA damage and cell death caused by exposure to iron (Lin et al. 2008; Wu, Xu, et al. 2023). Curcumin exhibits efficacy in the treatment of cancer through the regulation of oncogenes such as p53 and c‐myc, as well as transcription factors like NF‐kB and STAT‐3. Curcumin exhibits a synergistic impact when used with chemotherapy (Memarzia et al. 2022). Nanotechnology‐based formulations have been used to selectively deliver curcumin to tumors in recent years. This approach has the potential to enhance the effectiveness of curcumin for cancer chemoprevention and chemotherapy (Li et al. 2016).

Prior research has encompassed clinical investigations of curcumin's efficacy in treating various tumor‐related ailments, alongside preclinical examinations of other medications used in conjunction with curcumin for the treatment of NSCLC. Most clinical trials assessing curcumin's efficacy in NSCLC patients in the past decade included phase I and phase II trials. The safety and tolerability of curcumin in humans, therefore, are also evaluated in the present study. Nevertheless, there have not been any clinical trials that could determine the effectiveness of curcumin in treating NSCLC. Much of current research into the influence of curcumin on NSCLC consists of preclinical studies, mainly in vivo animal testing and cellular‐based assays. A meta‐analysis of the in vivo studies in this regard may result in valuable insight into curcumin's impact on NSCLC. It will help determine the potential necessity of fast‐tracking experimental interventions developed from preclinical to clinical settings (Pound et al. 2004). It was mainly focused on experimental studies in animals regarding possible curcumin effects in treating NSCLC, excluding all other studies involving curcumin in combination with other drugs. Our study aimed to conduct a comprehensive analysis of curcumin's effectiveness based on the existing literature.

**FIGURE 1:**
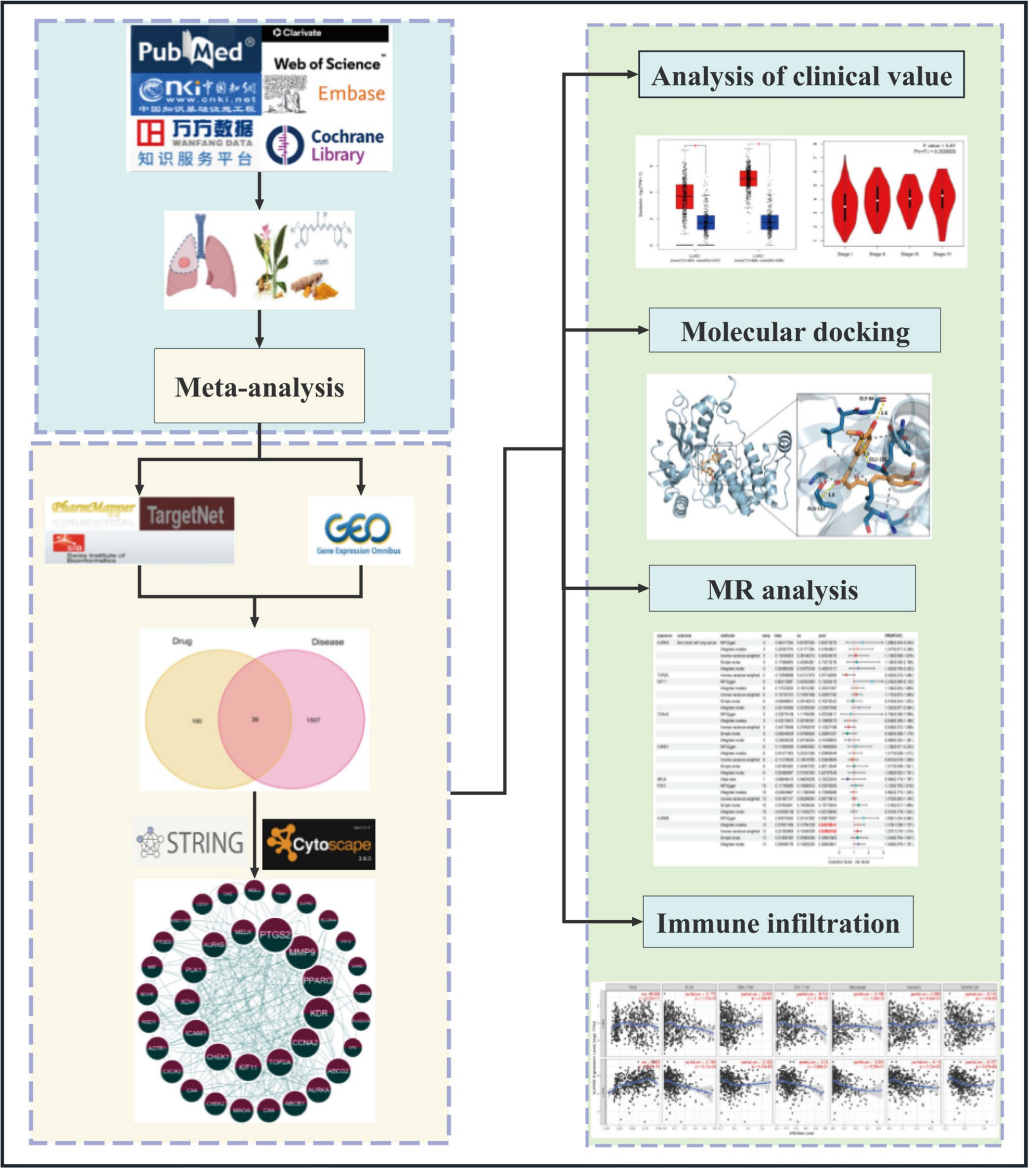
Workflow diagram.

The network pharmacology is a system‐level methodology used to analyze the molecular relationship between drugs and diseases to give an understanding of the biological network holistically (World Federation of Chinese Medicine Societies 2021). Mendelian randomization (MR) is a technique that employs single nucleotide polymorphism (SNP) as a means to evaluate the causal association between exposure variables and diseases (Davies et al. 2018). The study integrated research on evidence‐based medicine with network pharmacology analysis. It utilized meta‐analysis of animal studies to evaluate research findings in a sensible manner. Additionally, the network pharmacology analysis provided a comprehensive perspective on the mechanisms of medications. In order to uncover the mechanism of action, the MR analysis was performed to examine the causal link between major targets and NSCLC. Therefore, our study gathers and analyzes the available data from animal studies conducted before clinical trials. We investigate how curcumin functions in the treatment of NSCLC, aiming to establish a basis for potential clinical applications. The precise procedures are depicted in the workflow diagram (Figure 1).

## 2 Materials and Methods

In order to make suggestions for systematic review, this study used the Preferred Reporting Items (Page et al. 2021). The protocol utilizing the SYRCLE animal research tool (Hooijmans et al. 2014) has been officially recorded in PROSPERO (International Prospective Register of Systematic Reviews, registration number: CRD42024526189).

### 2.1 Meta‐Analysis Process

#### 2.1.1 Search Strategy

Data for this study was culled from six databases that researchers searched from their creation to December 2024 (Pubmed, EMBASE, Cochrane Library, Web of Science, CNKI, and Wan Fang Database). The following components made up the search strategy that was developed using the PICOS tool: (P) Lung Neoplasms; (I) curcumin; (S) Animals. The comprehensive search methodology is illustrated in Table 1, with Pubmed serving as an exemplar. The papers that were obtained were imported into the EndNote X9 software in order to remove any duplicate entries. Furthermore, supplementary publications that could be included were obtained by examining the references of these articles.

**TABLE 1:** Search strategy on PubMed.

Search	Pubmed
#1	Lung Neoplasms[MeSH Terms]
#2	(((((((((((((((((Lung Neoplasms[Title/Abstract]) OR (Pulmonary Neoplasms[Title/Abstract])) OR (Neoplasms, Lung[Title/Abstract])) OR (Lung Neoplasm[Title/Abstract])) OR (Neoplasm, Lung[Title/Abstract])) OR (Neoplasms, Pulmonary[Title/Abstract])) OR (Neoplasm, Pulmonary[Title/Abstract])) OR (Pulmonary Neoplasm[Title/Abstract])) OR (Lung Cancer[Title/Abstract])) OR (Cancer, Lung[Title/Abstract])) OR (Cancers, Lung[Title/Abstract])) OR (Lung Cancers[Title/Abstract])) OR (Pulmonary Cancer[Title/Abstract])) OR (Cancer, Pulmonary[Title/Abstract])) OR (Cancers, Pulmonary[Title/Abstract])) OR (Pulmonary Cancers[Title/Abstract])) OR (Cancer of the Lung[Title/Abstract])) OR (Cancer of Lung[Title/Abstract])
#3	#1 or #2
#4	curcumin[MeSH Terms]
#5	(((((curcumin[Title/Abstract]) OR (Turmeric Yellow[Title/Abstract])) OR (Yellow, Turmeric[Title/Abstract])) OR (Curcumin Phytosome[Title/Abstract])) OR (Phytosome, Curcumin[Title/Abstract])) OR (Diferuloylmethane[Title/Abstract])
#6	#4 or #5
#7	#3 and #6

#### 2.1.2 Inclusion and Exclusion Criteria

Our study necessitates the fulfillment of the following requirements: (1) It is necessary to assess and compare the effectiveness of curcumin as a therapy in an animal model of NSCLC, in comparison to a control group. (2) The measurement of tumor volume should be provided both before and after the treatment with curcumin, as an outcome measure. (3) Clearly stating the total number of animals in the control and treatment groups is of the utmost importance. (4) It is important to include the standard deviation (SD) or standard error of the mean (SEM) in the study results. (5) The research projects in the literature used standardized curcumin extracts or related pharmaceutical preparations (Izzo et al. 2016). (6) There are no limitations or restrictions regarding the dosage, method, or duration of administration.

Specific criteria were used to remove certain papers from the analysis: (1) Reviews, abstracts, comments, editorials, case reports, clinical trials, in vitro studies, and cell research were among the many study categories considered in this analysis. (2) We did not include papers in our meta‐analysis that did not employ an appropriate animal model of NSCLC. (3) Studies without a control group were excluded. (4) Studies with missing data were excluded. (5) Duplicate published studies were excluded. (6) Studies that did not use the desired outcome endpoint were excluded.

#### 2.1.3 Data Extraction

The included studies were analyzed, and data (authors, year of publication, animal type and strain, gender, age, test cell type, type of curcumin, dose, administration method, treatment duration, tumor volume, and tumor weight) were extracted into Microsoft Excel software by two independent researchers to create a database. Data were collected only for the highest dose when different doses of curcumin were used. We utilized Engauge Digitizer version 11.3 to extract data on tumor volume and tumor weight when the included articles gave results in graphical or pictorial form. The authors of these papers were contacted as needed in order to gather the appropriate data. The third researcher conducts a comparative analysis of the extracted data, addressing any inconsistencies identified within the dataset.

#### 2.1.4 Quality Assessment

Using the SYRCLE's risk of bias tool for animal experiments, two researchers independently evaluated the internal validity of the listed papers (Hooijmans et al. 2014). The evaluation checklist was comprised of the following six components: (1) selection bias: sequence generation, baseline characteristics, and allocation concealment; (2) performance bias: random housing and blinding of trial caregivers; (3) detection bias: random outcome assessment and blinding of outcome assessors; (4) attrition bias: incomplete outcome data; (5) reporting bias: selective outcome reporting; (6) other bias. The evaluation results of the 10 entries in this were ultimately evaluated on low risk, high risk, and not relevant criteria.

### 2.2 Network Pharmacology Exploration Process

#### 2.2.1 Prediction of Potential Targets of Curcumin

When predicting curcumin targets, researchers consulted the Swiss Target Prediction database, as well as PharmMapper and TargetNet (Daina et al. 2019; Wang et al. 2017; Yao et al. 2016). A search was conducted in the PubChem database to retrieve the SMILES and standard delay format (SDF) information for curcumin. After entering the SMILES data into Swiss Target Prediction, we used a probability threshold greater than 0 and specified that the species was humans to find possible curcumin drug targets. The SMILES data was also imported into TargetNet using a probability > 0 screening criterion to extract target information, which was then normalized in the UniProt protein database. We used PharmMapper to forecast curcumin targets by importing the SDF dataset, selecting “Human Protein Targets Only” as the species option, and screening the target data based on a Normalized Fit Score of 0.6 or higher. It ultimately came down to using UniProt to turn the target data into gene names.

#### 2.2.2 Acquisition and Processing of Gene Expression Omnibus (GEO) Data Sets

The R package “GEOquery” (Davis and Meltzer 2007) was utilized to download GSE18842 online from the GEO database as our study objects (including 46 NSCLC samples and 45 adjacent cancer tissues), which were normalized and integrated.

#### 2.2.3 Identification of Differentially Expressed Genes (DEGs)

To examine and filter DEGs, we employed the “limma” application (3.58.1). Adjusted *p*‐value < 0.05 and |log2(Fold Change) | > 1 were used to identify DEGs. We used the R packages “ggplot2” and “pheatmap.” to generate a Volcano plot.

#### 2.2.4 Weighted Gene Co‐Expression Network Analysis

To filter out genes and samples that did not fit, we used the “goodSamplesGenes” function in the WGCNA package. Plus, we used the R package “WGCNA” to construct the NSCLC gene co‐expression scheme. A suitable soft threshold was selected using the “pickSoftThreshold” function in order to bring the gene distribution into line with the scale‐free network. Using the WGCNA algorithm, we evaluated the connection between the clinical traits and the genes that make up the module's features. After determining which key module genes had the highest association coefficients with NSCLC, we analyzed them to see which ones were important.

#### 2.2.5 Venn Diagram Analysis

Drug target genes sourced from three databases underwent Venn diagram analysis using the R package “VennDiagram” (version 1.12), and the resulting union was then chosen as the predicted drug target. DEGs derived from the GSE18842 dataset were intersected with WGCNA key module genes, identifying intersection genes as key disease genes for NSCLC. These predicted drug target genes were further intersected with key disease genes to select genes for constructing the protein–protein interaction (PPI).

#### 2.2.6 Enrichment Analysis

The R packages “clusterProfiler” (4.10.1), “enrichment plot” (1.22.0), and “patchwork” (1.2.0) were utilized to conduct enrichment analyses, with a particular emphasis on the Gene Ontology (GO) and the Kyoto Encyclopedia of Genes and Genomes (KEGG) databases. Analyzing GO enrichment requires looking at cellular components (CCs), molecular functions (MFs), and biological processes (BP). To find pathways that were significantly linked to the gene (*p* < 0.05), we used the KEGG signaling pathway supplementation analysis.

#### 2.2.7 Construction of PPI Network

The website STRING (version 12.0) and the software Cytoscape (version 3.8.0) were used to predict and build the PPI network of diseases and medications. Subsequently, the hub genes were obtained by identifying critical subnetworks within the PPI network using the MCODE plugin within Cytoscape.

#### 2.2.8 Immune Infiltration Analysis of Hub Genes

Through the use of the TIMER website, we assessed the immune infiltration degree of hub genes in NSCLC.

#### 2.2.9 Mendelian Randomization

To delve deeper into the potential link between hub genes and NSCLC, a two‐sample MR study was used. Using the R package “TwoSampleMR” (0.5.8), we were able to obtain online hub gene and NSCLC SNPs. These SNPs served as instrumental variables (IVs) in our investigation into the potential causal relationship between hub genes and NSCLC outcomes as exposure factors. The study utilized SNPs that were screened after the influence of linkage disequilibrium was removed (*r* ^2^ = 0.1, kb = 100) and had a significance threshold of *p* < 1 × 10^−5^ as exposure factors so that there would be more SNPs to cover. Inverse variance weighted (IVW) was used as the analytical method for MR analysis in the study, with *p* < 0.05 of the IVW method considered to have a causal effect. Further analyses included an MR‐Egger regression to identify horizontal pleiotropy and a heterogeneity test using Cochran's *Q* statistic (Liu et al. 2023). SNPs were assessed for their strength using an *F* statistic; if *F* > 10, then SNPs were not surviving variables. Applying genetic variants as IVs, researchers utilized MR to evaluate the direct impact of exposure factors on outcomes, effectively eliminating any potential confounding factors and reverse causation.

#### 2.2.10 Clinical Value Analysis of Hub Genes

We used Gene Expression Profiling Interactive Analysis 2 (GEPIA2) to confirm the hub genes' expression levels (Tang et al. 2019). The data were acquired from the Cancer Genome Atlas (TCGA) database and the Genotype‐Tissue Expression Project (GTEx) database. The association between levels of hub genes and pathological grading of NSCLC was examined using the “Expression DIY” module in GEPIA2. |log2FC|Cutoff: 1, *p* Cutoff: 0.01.

#### 2.2.11 Molecular Docking

To obtain the 3D molecular structure of curcumin, we utilized PubChem. The Pymol 2.6 program was used to extract the structures of central targets from the Protein Data Bank (PDB) database, together with any nonessential ligands and water molecules, before treatment and hydrogenation. Docking was performed by predicting the binding pocket using DeepSite software (Jiménez et al. 2017). The AutoDock v1.5.6 software was used to achieve semiflexible docking (Jaghoori et al. 2016). The PILP website was used to analyze the docking results, which were imported into Pymol for visualization.

### 2.3 Statistical Analysis

Stata 15.0 software was applied for systematic review with meta‐analysis. To evaluate the treatment's effectiveness, we computed the standard mean difference (SMD) with 95% CIs, since all outcomes were continuous variables. According to Ruppar (2020), the *Q* statistic was used to detect heterogeneity inside a random effect model, and the *I* ^2^ findings were used to quantify it. Because *p* < 0.05, we may say that the difference is statistically significant. To determine the causes of heterogeneity, subgroup analysis was employed if *I* ^2^ > 50%. Results were more convincing when the meta‐analysis was rerun following the removal of one paper. The Egger bias test was used to determine if there was any publication bias.

Using R version 4.3.3, the network pharmacology investigations were analyzed statistically and visualized. For analyzing differential gene expression, one‐way ANOVA was employed. For two paired samples, the Wilcoxon paired rank sum test was employed. To do correlation analysis, Pearson's correlation tests were utilized. The GEPIA2 website was used to conduct a cooperative hypothesis test inquiry on pathology grading. The investigation of MR made use of five statistical methods: IVW, MR‐Egger, WME, SM, and WM. To account for the effect of linkage disequilibrium (*r* ^2^ = 0.1, kb = 100), a rigorous significance level of *p* < 1 × 10^−5^ was set up to establish a strong association between SNPs and exposure factors. We considered a difference to be statistically significant when *p* < 0.05.

## 3 Meta‐Analysis Results

### 3.1 Literature Selection

**FIGURE 2:**
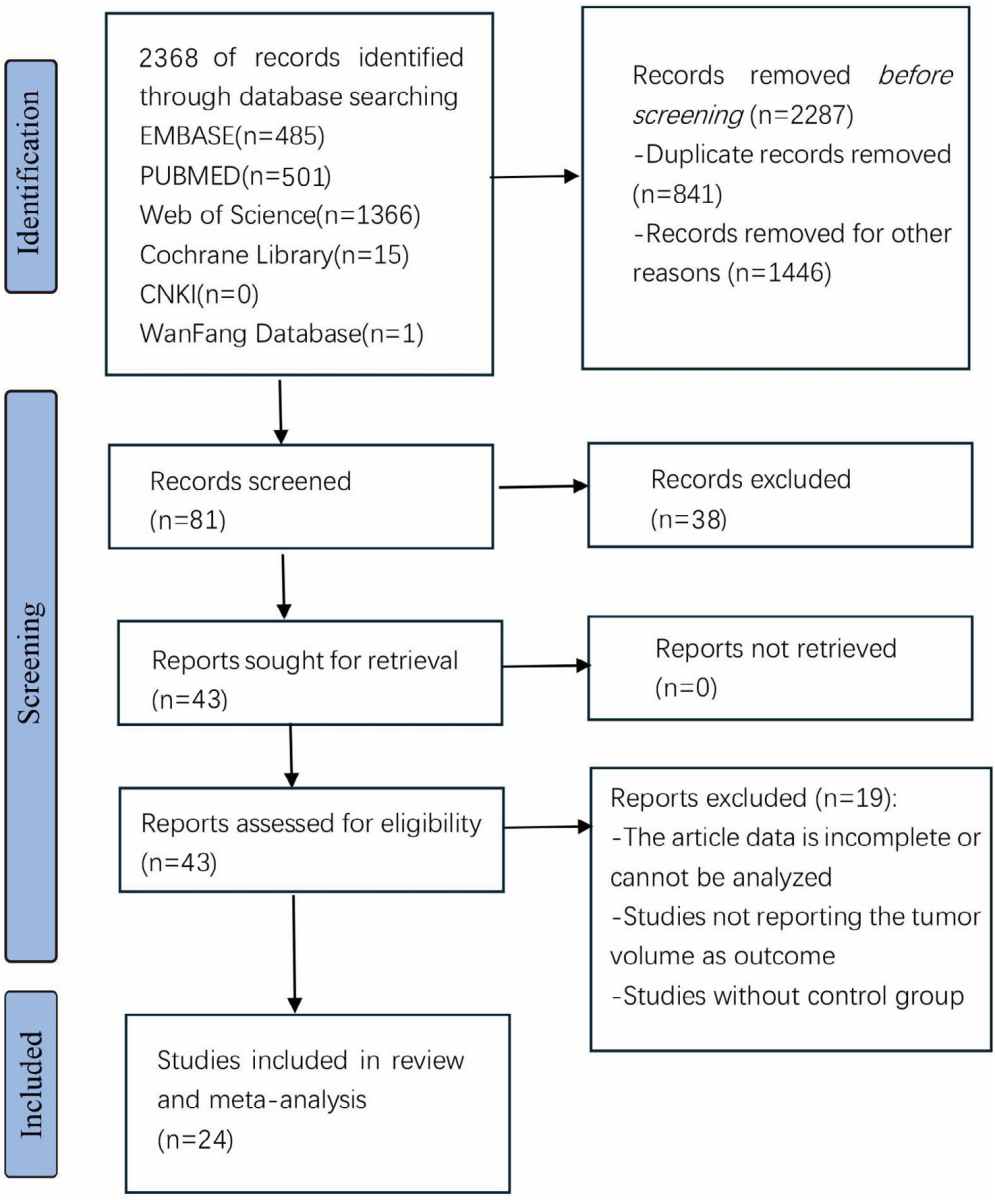
The study's screening workflow.

Initially, 2368 studies were retrieved, of which 841 were excluded due to duplicates and 1446 were excluded for various reasons such as cellular trials, reviews, abstracts, proposals, conference abstracts, incomplete experimental data, and the lack of experimental control groups. Following a full‐text evaluation, 24 studies were determined to be eligible for inclusion in this study, with Figure 2 depicting a flowchart of the literature selection process.

### 3.2 Study Characteristics

A total of 24 papers (Chen et al. 2016, 2014, 2020; Datta et al. 2013; Fan et al. 2014; Garbuzenko et al. 2014; Jiang et al. 2015; Lee et al. 2011; Li et al. 2024, 2016, 2013; Liu et al. 2016, 2017; Pi et al. 2022; Qin et al. 2024; Ranjan et al. 2016; Su et al. 2010; Tang et al. 2021; Wang et al. 2013, 2012; Wu, Chen, et al. 2023; Xu et al. 2024; Xu and Zhu 2017; Ye et al. 2015) investigating the effects of curcumin on animal models of NSCLC were included through systematic search and screening. All studies were conducted between 2010 and 2024. The papers examined the therapeutic benefits of curcumin in animal models of NSCLC. The animals examined in the publications were investigated using several mouse models. Specifically, seven papers utilized the Athymic nude mice model, eight papers employed the BALB/c nude mice model, seven papers utilized the C57BL/6J mice model, and two papers utilized the SCID mice model. The animal experiments utilized a diverse range of NSCLC cells, including A549 cells (*n* = 10) LLC cells (*n* = 7), H460 cells (*n* = 3), H1975 cells (*n* = 2), H358 cells (*n* = 1), ACC‐LC‐176 cells (*n* = 1), H1650 cells (*n* = 1), 801D lung cancer cells (*n* = 1), LK‐2 cells (*n* = 1), and A427 cells (*n* = 1). Out of the 24 researches conducted, 23 of them created tumor xenograft models of NSCLC by injecting tumor cells under the skin. Only one study used a different method, where they induced a carcinogenesis model by giving A549 human lung cancer epithelial cell suspensions directly to the lungs of mice via a catheter (Garbuzenko et al. 2014). Out of the 24 researches examined, 18 of them utilized curcumin for experimental intervention investigations. Among them, six papers employed curcumin unique nanoformulations specifically for animal experiments. In the study, a total of 392 participants were included, with 196 individuals assigned to the experimental group and another 196 individuals assigned to the control group. The doses of curcumin and its formulations that were given varied greatly, ranging from 2.5 to 1000 mg/kg. The treatment duration in the animal models of the cancers being studied showed considerable variation, ranging from 12 to 49 days. The primary findings of the research centered around the alterations in size and properties of the tumor tissues in NSCLC models. The data were provided as the average value with the corresponding SD. The specific characteristics were summarized in Table 2.

**TABLE 2:** Characteristics of 24 included studies.

Sequence	Author, year	Drug dosage form	Animal species	Gender	Age	Administration method	Drug dosage	Duration	Frequency	Tumor model induces cells	Outcome
1	Datta et al. (2013)	Curcumin	Athymic nude mice	Female	6 weeks old	Intravenous injection	50 mg/kg	28d	Once per 3 days	A549 cell, H358 cell, ACC‐LC‐176 cell	(1)(2)
2	Li et al. (2016)	CCM‐clns	C57BL/6J mice	Male	6 weeks old	Intraperitoneal injection	2.5 mg/kg	24d	Once per 2 days	LLC cell	(1)
3	Liu et al. (2016)	Curcumin	C57BL/6J mice	Female	5 weeks old	Intragastric administration	50 mg/kg	23d	Once a day	LLC cell	(1)(2)
4	Fan et al. (2014)	Curcumin	C57BL/6J mice	Female	NG	Intragastric administration	300 mg/kg	22d	Once a day	LLC cell	(1)
5	Li et al. (2013)	Curcumin	Athymic nude mice	Female	4 weeks old	Intragastric administration	1000 mg/kg	35d	Once a day	H1650 cell	(1)(2)
6	Wu, Chen, et al. (2023)	Curcumin	BALB/c nude mice	Male	NG	Intraperitoneal injection	50 mg/kg	30d	Once per 5 days	H1975 cell	(1)(2)
7	Chen et al. (2014)	Curcumin	Athymic nude mice	Male and female	NG	Intraperitoneal injection	60 mg/kg	28d	Once per 3 days	801D lung cancer cell	(1)(2)
8	Ye et al. (2015)	Curcumin	Athymic nude mice	Male	8 weeks old	Intragastric administration	300 mg/kg	15d	Once a day	H460 cell, A427 cell	(1)
9	Liu et al. (2017)	Curcumin	SCID mice	Male	6–8 weeks old	Intravenous injection	50 mg/kg	21d	Once per 5 days	A549 cell	(1)
10	Jiang et al. (2015)	Curcumin	BALB/c nude mice	Female	3–4 weeks old	Intraperitoneal injection	40 mg/kg	30d	Once per 2 days	A549 cell	(1)
11	Lee et al. (2011)	Curcumin	SCID mice	Male and female	6 weeks old	Intragastric administration	1000 mg/kg	42d	Once a day	H1975 cell, A549 cell	(1)
12	Su et al. (2010)	Curcumin	BALB/c nude mice	Female	NG	Intraperitoneal injection	45 mg/kg	30d	Once per 3 days	H460 cell	(1)
13	Garbuzenko et al. (2014)	Curcumin‐Janus nanoparticles	Athymic nude mice	Male and female	6–8 weeks old	Inhalation	2.5 mg/kg	28d	Twice a week	A549 cell	(1)
14	Ranjan et al. (2016)	Curcumin‐ER	Athymic nude mice	Female	4–6 weeks old	Subcutaneous injection	20 mg/kg	49d	Twice a week	A549 cell	(1)(2)
15	Xu and Zhu (2017)	Curcumin	Athymic nude mice	Male and female	4–6‐week‐old	Intragastric administration	100 mg/kg	28d	Once a day	H460 cell	(1)(2)
16	Tang et al. (2021)	Curcumin	C57BL/6J mice	Female	NG	Intraperitoneal injection	100 mg/kg	15d	Once a day	LLC cell	(1)
17	Pi et al. (2022)	Curcumin	BALB/c nude mice	Male and female	6 weeks old	Intraperitoneal injection	16 mg/kg	21d	Twice a week	A549 cell	(1)(2)
18	Wang et al. (2012)	Liposomal curcumin	C57BL/6J mice	Female	6 weeks old	Intravenous injection	10 mg/kg	14d	Once a day	LLC cell	(1)
19	Wang et al. (2013)	Curcumin/MPEG‐PCL micelles	C57BL/6J mice	Female	6–8 weeks old	Intravenous injection	5 mg/kg	20d	Once per 5 days	LLC cell	(1)(2)
20	Chen et al. (2016)	Curcumin	C57BL/6J mice	Male and female	NG	Intraperitoneal injection	40 mg/kg	25d	Once per 2 days	LLC cell	(1)(2)
21	Chen et al. (2020)	Curcumin	BALB/c nude mice	Female	6–8 weeks old	Intravenous injection	2.45 mg/kg	12d	Once per 3 days	A549 cell	(1)(2)
22	Qin et al. (2024)	Curcumin	BALB/c nude mice	Male	5 weeks old	Intraperitoneal injection	40 mg/kg	21d	Once a day	A549 cell	(1)(2)
23	Xu et al. (2024)	Curcumin	BALB/c nude mice	Male	4 weeks old	Intragastric administration	50 mg/kg	28d	Once a day	LK‐2 cell	(1)(2)
24	Li et al. (2024)	CUR SLNs	BALB/c nude mice	Male and female	9–10 weeks old	Intravenous injection	5 mg/kg	21d	Once per 3 days	A549 cell	(1)

*Note*: Outcomes: (1) Tumor volume; (2) tumor weight.

Abbreviations: CCM: curcumin, CCM‐clns: Curcumin catanionic lipid nanosystems, CUR SLN: curcumin solid lipid nanoparticle, Curcumin‐ER: hybrid curcumin nanoformulation.

### 3.3 Study Quality

**FIGURE 3:**
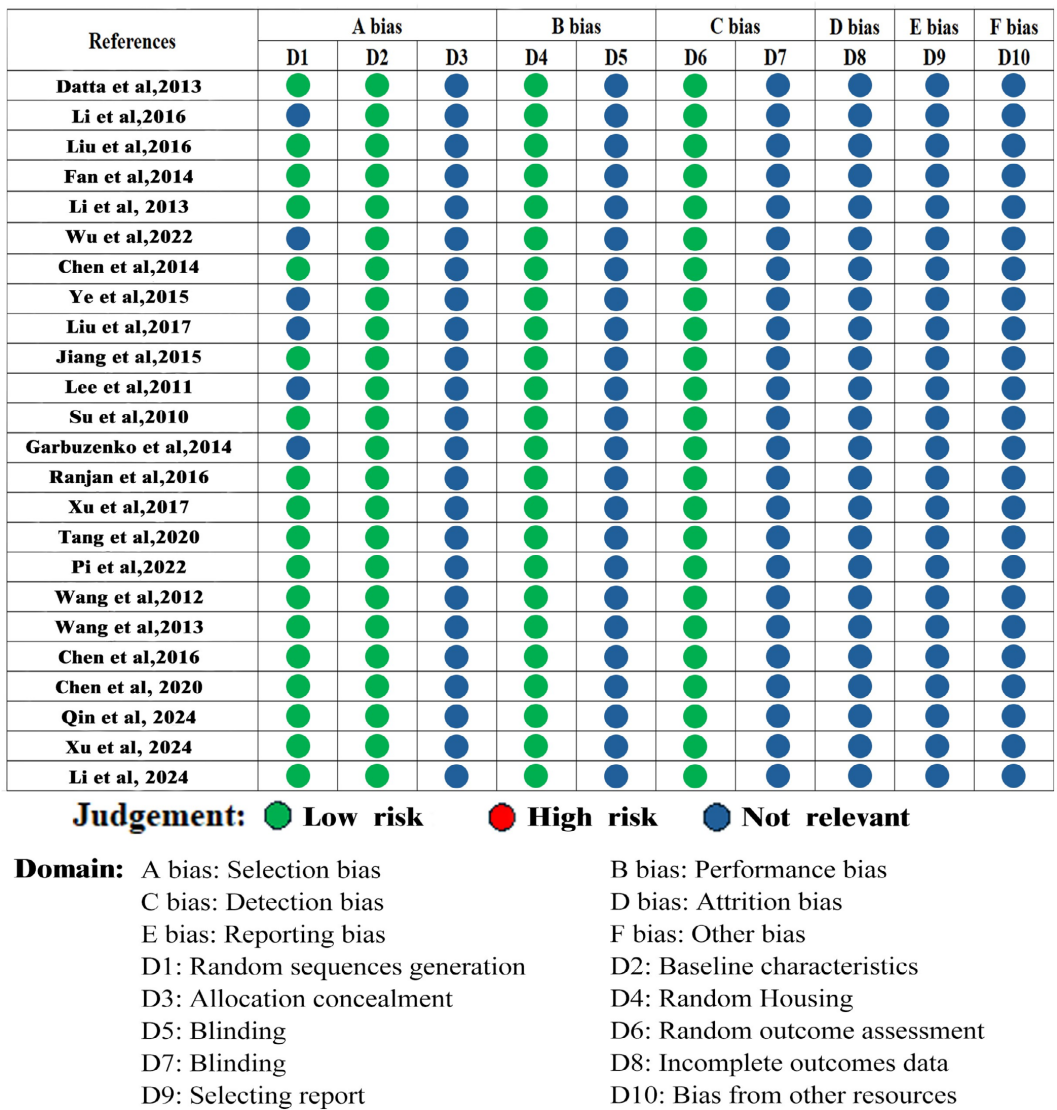
SYRCLE's risk of bias assessment for included studies.

Research involving animals was evaluated using the SYRCLE's risk tool (Figure 3). Blinding trial caregivers and researchers, blind outcome assessors, missing outcome data, and explaining how selective result reporting or allocation concealment were not addressed in any of the 24 trials that were statistically reviewed. In all, 18 experiments, or 75%, made use of randomized sequences in their design. The baseline features of animals, including their species, age, and gender, were described in detail in 18 studies. While the body weight was reported in six experiments, the age of the mice was not. There were no studies omitted because of poor quality, indicating that the overall research quality was adequate.

### 3.4 Tumor Volume

**FIGURE 4:**
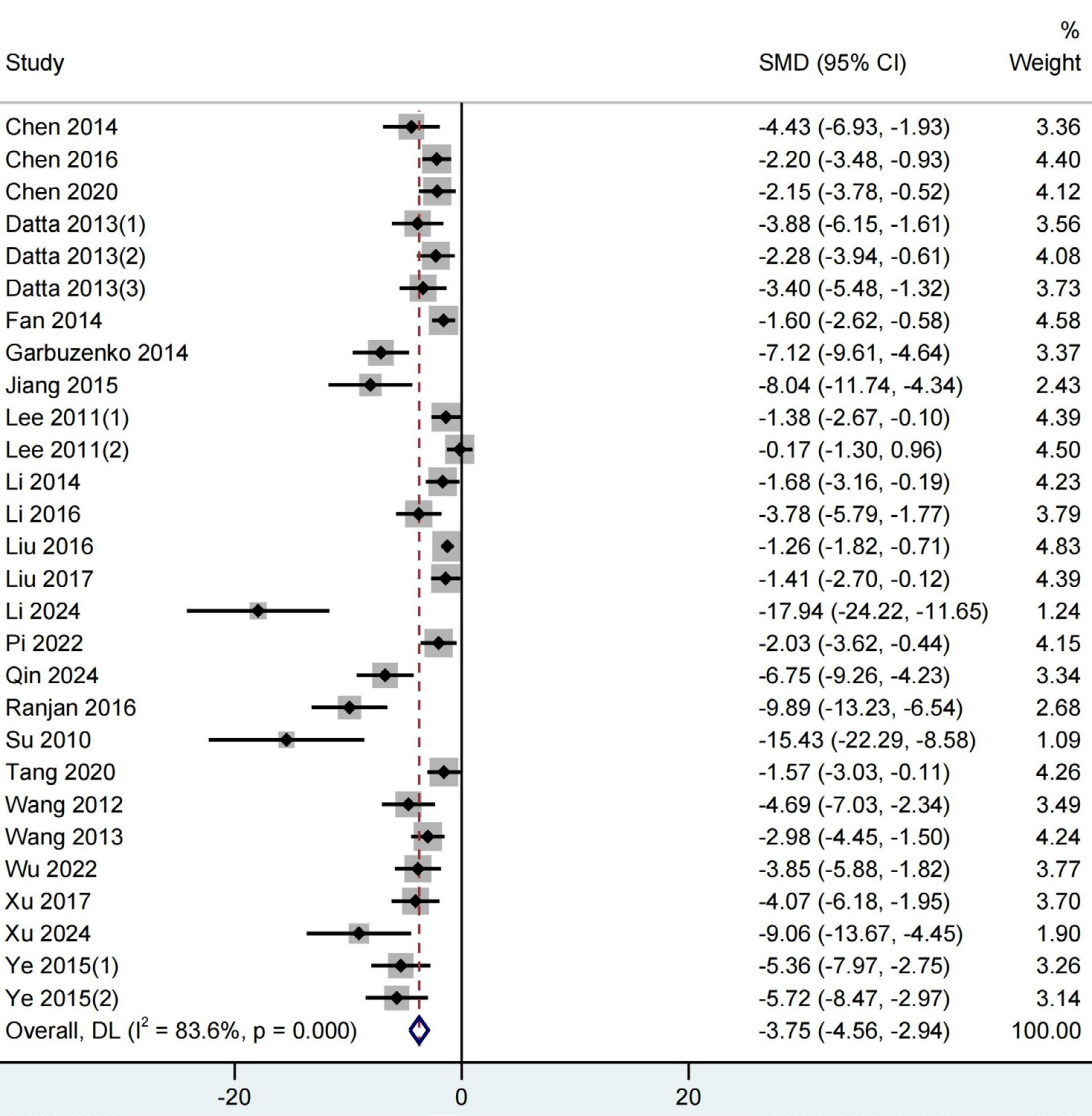
Forest plot of curcumin effects on tumor volume of NSCLC. Heterogeneity: *τ* ^2^ = 3.447, *χ* ^2^ = 164.70; df = 27; *p* < 0.001; *I* ^2^ = 83.6%. Test for overall effect: *Z* = −9.085 (*p* < 0.001). Weights are from random‐effect model.

The change in tumor volume directly reflects the entire process from formation to development. In our study, tumor volume was used as the primary outcome based on the research content and data integrity of the included literature. The secondary outcome was tumor weight (Figure S1). The findings from the meta‐analysis on the impact of curcumin on NSCLC, as presented in Figure 4, used a random effect model. This analysis specifically examines the outcomes related to tumor volume. It was observed across 24 included studies (Chen et al. 2016, 2014, 2020; Datta et al. 2013; Fan et al. 2014; Garbuzenko et al. 2014; Jiang et al. 2015; Lee et al. 2011; Li et al. 2024, 2016, 2013; Liu et al. 2016, 2017; Pi et al. 2022; Qin et al. 2024; Ranjan et al. 2016; Su et al. 2010; Tang et al. 2021; Wang et al. 2013, 2012; Wu, Chen, et al. 2023; Xu et al. 2024; Xu and Zhu 2017; Ye et al. 2015), comprising 392 animals, that curcumin significantly reduced tumor volume of NSCLC (*p* < 0.001) (SMD = −3.75; 95% CI: −4.56 to −2.94). These results suggest that all treatments variably inhibited tumor volume growth, indicating curcumin's potential inhibitory effects on NSCLC. The heterogeneity of the included studies was high (*I* ^2^ = 83.6%).

### 3.5 Subgroup Analysis for Tumor Volume

Subgroup analysis was conducted to identify sources of heterogeneity, examining factors such as curcumin administration dose, method, duration, and animal gender. The majority of the studies employed xenograft models. The analysis indicated that the administration dose, method, duration, and animal gender could be potential sources of heterogeneity (Figure S2).

### 3.6 Sensitivity Analysis

**FIGURE 5:**
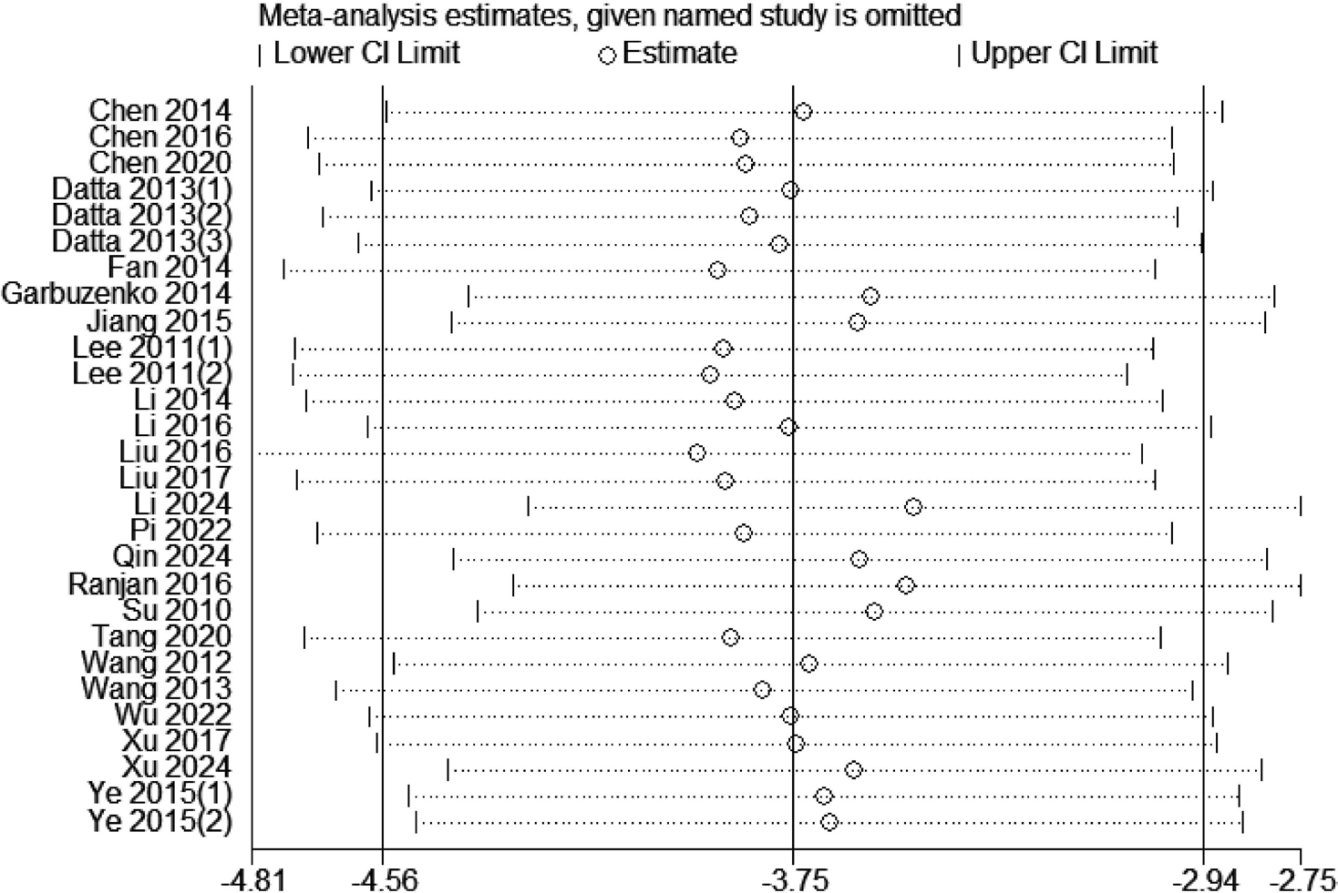
Sensitivity analyses.

The dependability of the positive test results in the animal experimental study on curcumin's effects on NSCLC was evaluated using a sensitivity analysis. This was done by examining any variations in tumor volume. Based on the analysis, it was found that there were no notable changes in the overall impact of the study findings, as shown in Figure 5.

### 3.7 Publication Bias

**FIGURE 6:**
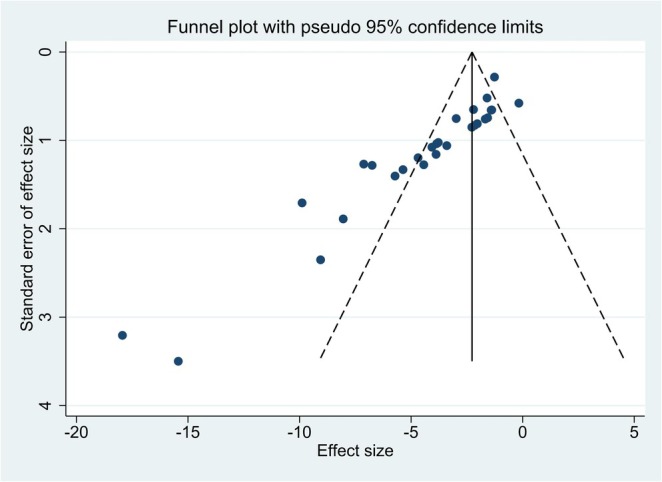
Publication bias.

Results from Egger's test, which were presented in Table 3 and Figure 6, suggest that publication bias exists in the meta‐analysis.

**TABLE 3:** Assessment of publication bias for the impact of curcumin.

Outcome	Egger's regression test		
	95% CI	*t*	*p*
Tumor volume	−5.29 to −3.45	−9.79	< 0.001

Abbreviation: CI: confidence interval.

## 4 Network Pharmacology Results

### 4.1 Curcumin Target Gene Prediction and Identification of Hub Genes in NSCLC

**FIGURE 7:**
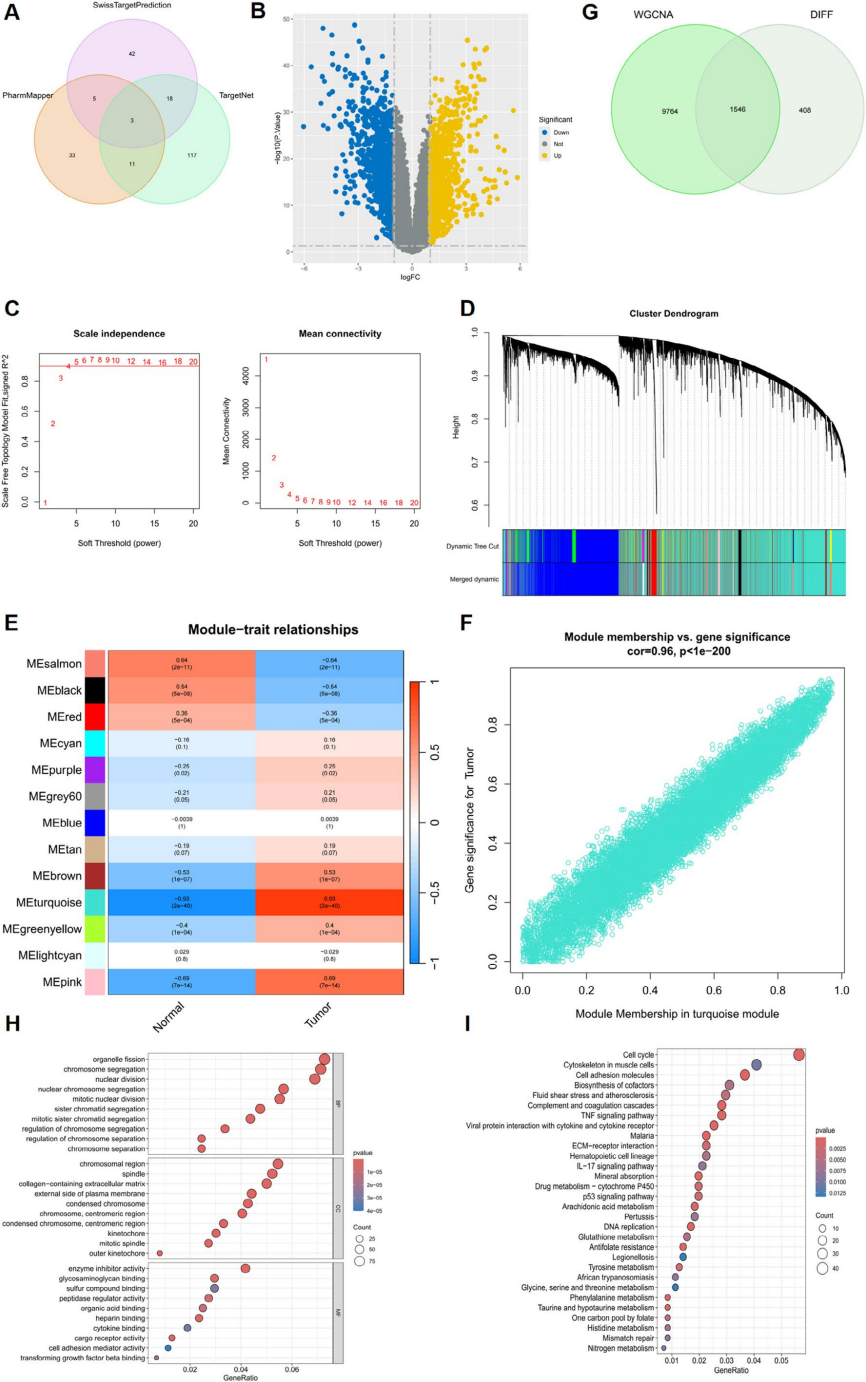
Curcumin target gene prediction and identification of hub genes in NSCLC. (A) Venn diagram of three databases predicting curcumin targets. (B) Volcano plot of DEGs. (C) Scale independence and mean connectivity. (D) Cluster dendrogram of coexpressed genes in NSCLC. (E) Correlation heatmap of module characteristic genes and phenotypes. (F) Correlation between turquoise module and NSCLC. (G) Venn diagram of hub genes in NSCLC. (H) GO Enrichment analysis. (I) KEGG enrichment analysis.

We extracted 68 predicted genes from the Swiss Target Prediction database. The PharmMapper database provided us with 52 predicted genes. Additionally, the TargetNet database yielded 174 predicted targets. A Venn diagram analysis (Figure 7A) of the predicted targets from these three databases revealed 229 potential targets for curcumin (Table S1). The set of 229 target genes underwent further study, which included KEGG signaling pathway analyses and GO enrichment studies (Figure S3).

The GSE18842 dataset, encompassing transcriptomic data from 46 NSCLC patients and 45 adjacent cancer tissues, was acquired from the GEO database and normalized. Using the limma package for differential expression analysis, DEGs were identified between NSCLC patients and adjacent non‐cancerous tissues. A volcano plot (Figure 7B) displayed 876 significantly up‐regulated and 1089 significantly down‐regulated DEGs, based on an adjusted *p*‐value < 0.05 and |log2 (Fold Change)| > 1. The WGCNA package simultaneously found gene modules that were coexpressed in NSCLC tumor tissues. To choose the suitable soft threshold that adheres to the scale‐free network standard, the “pickSoftThreshold” function from the WGCNA package was utilized. Figure 7C shows that a soft threshold of 4 was chosen after establishing a power range of 1–20 and *R* ^2^ > 0.9. Figure 7D–F show the 13 co‐expressed gene modules; each color represents a different module. The turquoise module, which contains 11,449 genes, was found to be the key module linked to NSCLC (*r* = 0.93, *p* = 2e−40). The genes in the key module were compared with DEGs through Venn diagram analysis, yielding 1546 intersecting genes (Figure 7G). Then, GO and KEGG analyses were performed on these. As evident in Figure 7H, the GO analysis revealed that BP was highly enriched (*p* < 0.05) for disease genes related to organelle fission, nuclear division, and chromosome segregation. Enrichment was most concentrated in the CC‐containing spindle, chromosomal region, and extracellular matrix that contains collagen. Enzyme inhibitor, glycosaminoglycan binding, and peptidase regulator activities were the primary MF enrichment locations. Figure 7I demonstrates that the illness genes were primarily linked to the Cell cycle, Cell adhesion molecules, and TNF signaling pathway by KEGG pathway enrichment analysis (*p* < 0.05).

### 4.2 Screening of Hub Genes for Disease and Drug Target Intersection by PPI Network

**FIGURE 8:**
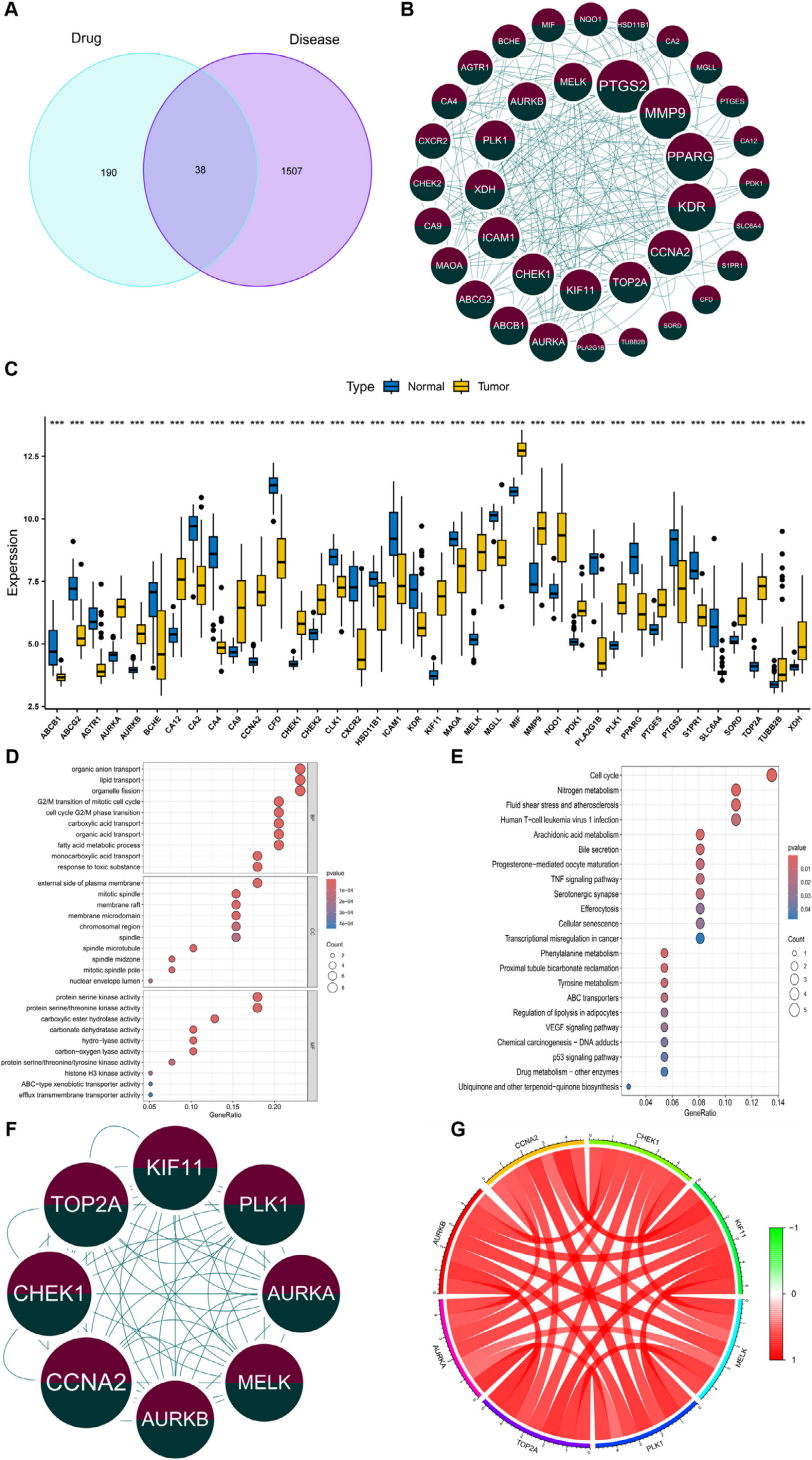
Screening of hub genes for disease and drug target intersection by PPI network. (A) Venn diagram of disease and drug targets. (B) PPI network of 38 intersection genes. (C) Differential expressions of 38 intersection genes in normal and tumor tissues (*p < 0.05, **p < 0.01, ***p < 0.001). (D) GO enrichment analysis of 38 intersecting genes. (E) KEGG enrichment analysis of 38 intersecting genes. (F) PPI network of eight hub genes. (G) Correlation network of eight hub genes.

To construct the PPI network, 38 intersection genes were obtained by intersecting 1546 key disease genes and 229 drug target genes (Figure 8A). Using Cytoscape (3.8.0) and the STRING online database (version 12.0), we examined the PPI of intersection genes (Figure 8B). The MCODE plugin in Cytoscape was used to identify critical subnetworks (Table S2). Figure 8C shows the results of an analysis of the 38 genes' differential expression in the tumor and normal patient groups using the limma package. The results showed significant differences. Specifically, the expressions of TUBB2B, XDH, MMP9, SORD, CHEK2, MIF, PLK1, CCNA2, KIF11, AURKA, CHEK1, AURKB, TOP2A, MELK, NQO1, PTGES, CA12, PDK1, and CA9 were increased in the tumor group. Conversely, the expressions of BCHE, MAOA, CA2, SLC6A4, ABCG2, PPARG, CFD, CA4, PLA2G1B, CXCR2, PTGS2, ABCB1, S1PR1, AGTR1, KDR, ICAM1, MGLL, CLK1, and HSD11B1 were decreased in the tumor group compared to the normal group. Figure 8D shows that the 38 genes that were found to be involved in the intersection were enriched in GO enrichment analysis related to biological functions (such as organic anion transport, organelle fission, and lipid transport), cellular locations (such as the outside of the plasma membrane, the mitotic spindle, and the membrane raft), and MFs (such as protein serine kinase activity and protein serine/threonine kinase activity). Figure 8E shows the results of the KEGG pathway enrichment analysis, which revealed that these genes were highly enriched in pathways related to nitrogen metabolism, fluid shear stress, atherosclerosis, and the cell cycle. The PPI analysis identified eight hub genes in critical subnetworks, namely CCNA2, AURKB, MELK, CHEK1, AURKA, PLK1, TOP2A, and KIF11 (Figure 8F). Subsequent analysis of these eight hub genes using the “Pearson” correlation test on the GSE18842 dataset showed a positive correlation, indicating strong interactions among them (Figure 8G).

### 4.3 Clinical Value Analysis of Hub Genes

**FIGURE 9:**
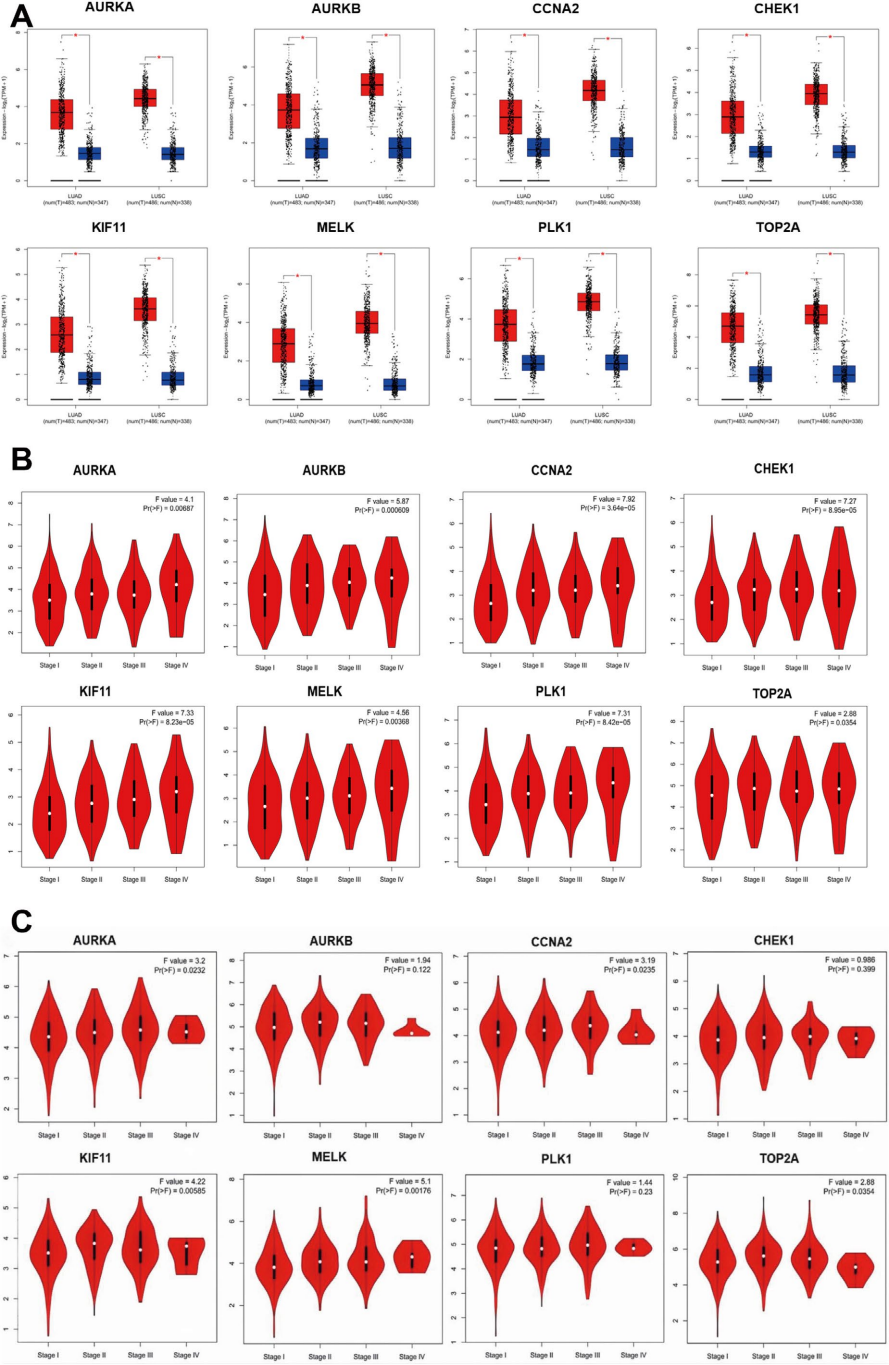
Clinical value analysis of hub genes. (A) Differential expression analysis of hub genes in NSCLC and normal tissues based on GEPIA2 database; (B) correlation between hub gene expressions and pathological grades of LUAD based on the GEPIA2 database; (C) correlation between hub gene expressions and pathological grades of LUSC based on the GEPIA2 database.

We searched the GEPIA2 database for evidence of the hub genes' diagnostic importance in clinical practice. In Figure 9A, we can see that compared to normal samples, tumor tissue samples from NSCLC patients had considerably higher levels of hub gene expression (*p* < 0.01). To investigate whether hub genes are associated with NSCLC pathological grades, we used the GEPIA2 database. Hub genes were related to tumor pathological grades of lung adenocarcinoma (LUAD) in LUAD tissues, as shown in Figure 9B (*p* < 0.05). There is a direct correlation between the severity of LUAD and the levels of expression of the eight genes mentioned in tumor tissues. In Figure 9C, AURKA, CCNA2, KIF11, MELK, and TOP2A showed a strong correlation with tumor pathological grades of LUSC in the LUSC tissues (*p* < 0.05). The aforementioned findings indicate that hub genes possess significant clinical diagnostic utility for NSCLC, particularly, LUAD.

### 4.4 Immune Infiltration Analysis of Hub Genes

We investigated the link between immune cell invasion and eight hub genes using the TIMER website. The eight hub genes were found to be negatively correlated with immune cell infiltration according to research. According to Figure S4, this indicates that the eight hub genes might impede the immune response to malignancies and make it easier for NSCLC to evade the immune system.

### 4.5 Molecular Docking

**FIGURE 10:**
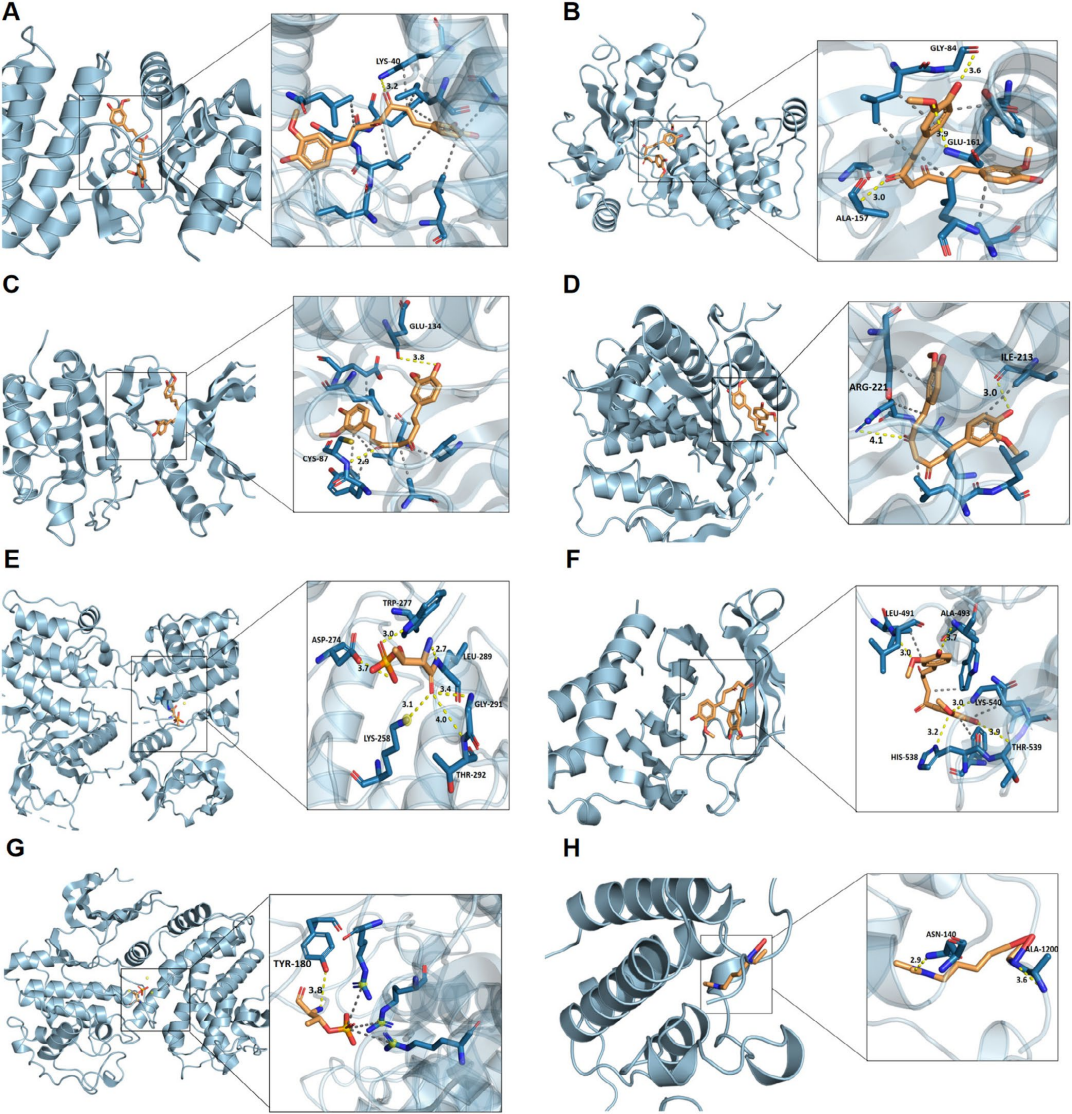
Molecular docking of curcumin and hub genes. (A–H) Molecular docking was performed with MELK, AURKB, CHEK1, KIF11, AURKA, PLK1, CCNA2, TOP2A, and curcumin; yellow dashed lines indicate hydrogen bonds.

The results of molecular docking indicate that the binding energy between the hub genes and curcumin is less than −5.0 kcal/mol, suggesting a strong binding ability between curcumin and the action targets. PyMol software was used to visualize the results (Figure 10A–H). The binding energies for MELK, AURKB, CHEK1, KIF11, AURKA, PLK1, CCNA2, and TOP2A were −9.6 , −8.6, −8.4, −7.9, −7.7, −7.3, −6.2, and −6.0 kcal/mol (Table S3). Molecular docking suggests that curcumin may exert its role by acting on hub genes.

### 4.6 Prognostic Value of MR Analysis of Hub Genes

**FIGURE 11:**
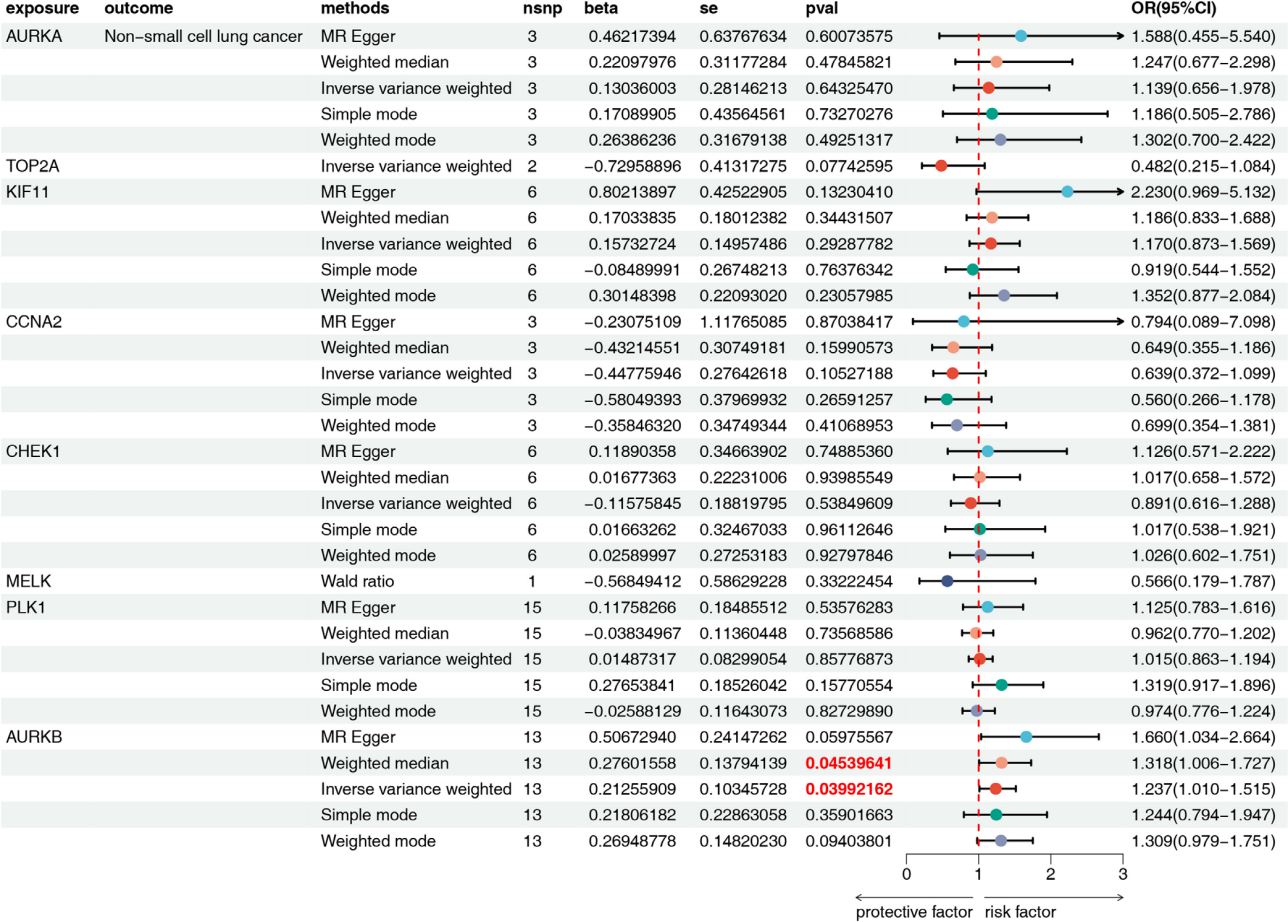
Forest plot of MR results of hub genes and NSCLC.

MR Analysis was selected to assess the causal relationship between hub genes and NSCLC. By calculating the *F* value of SNPs (*F* > 10), it was shown that SNPs are not weak IVs (Table S4). In this study, IVW was used as the main analysis method for MR analysis. Analysis results show that a causal relationship exists between the AURKB gene and the increase in risks of NSCLC (OR = 1.237; 95% CI = 1.010–1.515; *p* = 0.03992162); however, no significant causal relationships were identified for other hub genes with NSCLC. These findings are visualized in a forest plot (Figure 11). Tests for heterogeneity and pleiotropy were conducted using Cochran's *Q* statistic and MR‐Egger regression, respectively, with both methods indicating no significant findings (*p* > 0.05). The absence of significant heterogeneity and pleiotropy in other hub genes is attributed to insufficient SNPs in MELK (Table S5). Further analysis of AURKB through the GEPIA2 database revealed that high AURKB expression correlates with shorter overall survival (OS) and progression‐free survival (PFS), supporting its identification as a risk factor for NSCLC (Figure S5).

## 5 Discussion

Curcumin, the bioactive compound found in the edible spice turmeric, was the traditional medicine in China (Ammon and Wahl 1991). Preclinical studies have shown that curcumin has therapeutic promise for various human disorders, particularly, in the treatment of multiple malignancies (Aggarwal and Harikumar 2009). At present, the chemotherapeutic medicines most commonly employed for lung cancer treatment, namely paclitaxel, carboplatin, and gemcitabine, exhibit restricted effectiveness and frequently induce significant adverse reactions. Hence, it is imperative to do research on novel therapy alternatives that are both more efficacious and less harmful. Curcumin has the ability to block the G2/M cell cycle phase, induce programmed cell death, and reduce cell growth in NSCLC. Because its effectiveness in tumor formation and tumor growth is very promising for tumor size reduction, this makes it a good target for conducting studies on lung cancer (Giordano and Tommonaro 2019; Mansouri et al. 2020; Wang, Song, et al. 2019). Addressing the pathogenesis and therapeutic options for NSCLC and identifying relevant targets remain critically urgent.

Several recent clinical trials have tested its effectiveness when taken in combination with other drugs. One such trial, known as NCT 02321293, focused on using CURCUVivaTM and TKI together to treat advanced NSCLC in patients with EGFR mutations. This 55‐patient trial was part of a larger effort to determine whether or not end‐stage NSCLC patients may safely and effectively undergo curcumin combination therapy (Esfahani et al. 2019). The clinical trial (JPRN‐UMIN 000006892) examined the safety and practicality of using a combination therapy of erlotinib and curcumin nanoparticles to treat patients with advanced or recurrent NSCLC (Tang et al. 2022). The majority of ongoing clinical trials primarily focus on assessing the human safety and tolerability of curcumin. Clinical trials using curcumin on human beings to establish the therapeutic advantage of such a compound in the treatment of NSCLC have never been conducted. The reliability of the findings related to NSCLC on animal studies needs to be gauged through the systematic review and meta‐analysis, as this will help facilitate details about the discrepancies that existed between pre‐clinical and clinical trial results. Additionally, it also helps in designing clinical trials (Vesterinen et al. 2014). The effect of curcumin on NSCLC treatment in animal studies was the subject of a meta‐analysis. Furthermore, the network pharmacology analysis was carried out to delve deeper into the particular targets of curcumin in NSCLC and lay the theoretical groundwork for its practical application in treating NSCLC.

According to trial statistics, the efficacy of curcumin in preventing tumorigenesis, inhibiting spread, or reducing tumor size was evaluated. Analysis of independent studies, using random effects models, showed that curcumin and its derivatives variably reduced tumor volume (Figure 1) and tumor weight in test animals (Figure S1). These findings suggest a potential inhibitory effect of curcumin on NSCLC cell growth. The heterogeneity of the included studies was high. We conducted a subgroup analysis to investigate the sources of heterogeneity. In the meta‐analysis of animal experiments, high heterogeneity may arise from multiple factors. Variations in curcumin dosage, administration methods, duration of treatment, and sex of mice are among the contributors to this high level of heterogeneity (Figure S2). Additionally, differences in mouse strains (Athymic nude, C57BL/6J, BALB/c nude, SCID) may contribute to variability. For instance, in tumor research, C57BL/6 mice and BALB/c mice exhibit distinct immune responses to tumor cells, which can result in variability in experimental outcomes (Okeoma et al. 2009). Furthermore, individual characteristics such as age and weight of the animals can also influence the results obtained. The differences in weight and age of the mice may reflect the nutritional status and health level of the mice, which can affect their susceptibility to the disease model and lead to heterogeneity in the test results.

There have been no major adverse effects recorded in the evaluation of oral and parenteral (IP and IV) administrations in both model animals and human clinical studies (Heger et al. 2014; Nelson et al. 2017), confirming their safety and feasibility with no serious adverse reactions reported. Various administration methods demonstrated significant antitumor effects by attenuating tumor growth and reducing tumor mass. Oral administration is favored due to its ease of compliance and non‐invasiveness (Du et al. 2013; Hussain et al. 2017). Recently, numerous novel drug formulations, including curcumin nanoparticles, have been developed and examined (Silvestre et al. 2023). This exploration of diverse administration methods in animal models paves the way for investigating clinical administration strategies for curcumin and developing new pharmaceutical preparations.

The safety, tolerability, and lack of toxicity of curcumin have been extensively studied in human trials. These trials have shown that curcumin is well‐tolerated when taken orally at dosages of up to 2 g/kg in animals and 1 g/kg in humans, respectively (Lao et al. 2006). In the 30‐day toxicity study, rats received curcumin granules at doses of 5, 2, and 0.5 g/kg/d on a daily basis. The study found no significant impacts or variations in behavior, physiology, and pathology among the rats (Fu et al. 2021). The maximum safe dosage used in human clinical trials exceeds 120 mg/m^2^ (Storka et al. 2015). Current toxicity assessments of curcumin have demonstrated few toxicological effects.

The therapeutic regimens investigated in the study of animal tumor models ranged from 12 to 49 days. Tumor volume growth was reduced across various administration courses without evidence of drug accumulation or toxicity in the model group. In a different study, long‐term (3 months), low‐dose (0.25 ~ 0.5 μM) curcumin administration showed an inhibitory effect on the invasive and metastatic potential of NSCLC cells (Smagurauskaite et al. 2020). Chemoprevention trials indicate that prolonged use of single agents might foster clonal evolution and produce treatment‐resistant subclones, potentially diminishing the effectiveness of conventional chemotherapy (Potter 2014). Consequently, awareness of the long‐term effects of drug use would enable it to be possible to calculate the potential detrimental influences on the efficacy of later treatments.

The pharmacological mechanism of curcumin in the treatment of NSCLC is still unclear. The inhibitory effect of curcumin on NSCLC resistance may be related to its antitumor mechanism of action (Xie et al. 2022). Meta‐analysis can summarize and analyze the results of a large number of observational studies, but it may be affected by confounding factors inherent in the observational studies themselves, making it difficult to establish a clear causal relationship. Network pharmacology can depict the complex biomolecular networks related to diseases and show the interactions between genes, proteins, metabolites, and so forth, identifying potential key nodes and pathways of diseases. There have already been network pharmacology studies on the treatment of osteoarthritis, periodontitis, and the synergistic treatment of hepatocellular carcinoma cells with curcumin (Guzmán‐Flores et al. 2023; Jin et al. 2024; Wang et al. 2024). However, the network pharmacology of curcumin for treating NSCLC remains unstudied. Combining the meta‐analysis, MR, and network pharmacology can integrate multi‐source data such as traditional experimental research data, biomolecular network data, and genetic data organically. This approach can mine data information from different angles and levels, providing more comprehensive data support for research.

From Swiss Target Prediction, PharmMapper, and TargetNet databases, 229 target genes of curcumin were screened out. Disease hub genes were identified through differential expression analysis and WGCNA on the GEO dataset GSE18842. Key module genes identified by WGCNA were cross‐referenced with DEGs to determine 1546 disease hub genes. These were further intersected with predicted curcumin targets, identifying 38 potential action targets for curcumin in the treatment of NSCLC. The 38 genes that were found by GO enrichment analysis primarily have roles in molecular processes and cellular localization. Take protein serine kinase and protein serine/threonine kinase activities as examples. These genes may have a role in regulating the cell cycle, since this was the most abundantly represented pathway in KEGG. A number of mechanisms, including curcumin's effects on the EGFR and TLR4/MyD88 pathways and its synergistic interactions with downstream regulators of the cell cycle and EMT, contribute to its inhibitory effects on NSCLC cell proliferation and metastasis (Zhang et al. 2019). Curcumin inhibits the NF‐κB/MMP pathway in NSCLC, which in turn decreases adiponectin production, impacts cell proliferation, and ultimately causes cell death (Tsai et al. 2015). Our findings align with prior research.

PPI networks were constructed using intersection genes, from which 8 core targets of curcumin for NSCLC treatment were identified using the MCODE plug‐in. These targets demonstrated high expression in NSCLC tissues according to the GEPIA2 database. The eight hub genes showed elevated expression levels in both LUAD and LUSC tumor tissues. Moreover, all these eight hub genes in LUAD were closely associated with tumor grading, although in LUSC, some were related to the grading of the tumor. These results indicated that these 8 core targets are significant in NSCLC, especially LUAD. The analysis of immune infiltration revealed a negative correlation between these core targets and most immune cell types in tumor tissues, such as B cells, CD4+ T cells, and macrophages. Previous research has highlighted the crucial role of B cells (Wang, Liu, et al. 2019), T cells (Wu, Yuan, et al. 2023), and macrophages (Sedighzadeh et al. 2021) in antitumor immunity within lung cancer tissues. Our findings suggest that these core targets may inhibit immune cell infiltration and facilitate NSCLC's evasion of the body's antitumor immune response. Curcumin is recognized as an immunomodulator, influencing the activation of T cells, B cells, macrophages, and neutrophils in vivo (Jagetia and Aggarwal 2007). Notably, low doses of curcumin have been shown to induce CD8+ T cell‐mediated antitumor immune responses in mice bearing LUAD LUADs (Luo et al. 2011). In conclusion, our study reveals that curcumin may exert antitumor effects by modulating immune cells in human NSCLC.

Subsequent molecular docking studies indicated that the eight target proteins (MELK, AURKB, CHEK1, KIF11, AURKA, PLK1, CCNA2, and TOP2A) exhibit strong binding affinity for curcumin. Therefore, it is hypothesized that curcumin targets the expression and/or function of these core proteins, enhancing its antitumor capabilities and therapeutic potential in NSCLC.

A correlation study has revealed a positive connection between the eight hub genes, which have a significant impact on the development of NSCLC. The proteins encoded by CCNA2 are part of a highly conserved family of cell cycle proteins crucial for cell cycle regulation and closely associated with LUAD tumorigenesis (He et al. 2024). AURKB regulates chromosome alignment and segregation during mitosis and meiosis by interacting with microtubules. Aurora B kinase targeting enhances BIM‐ and PUMA‐mediated apoptosis, which in turn prevents and counteracts EGFR inhibitor resistance in NSCLC (Tanaka et al. 2021). The activation of calcium‐binding, non‐membrane trans‐protein tyrosine kinase by MELK is associated with various activities such as protein serine/threonine kinase activity, autophosphorylation of proteins, and cell proliferation (Tang et al. 2023). By interacting directly with NSCLC cell Smad family members (Smad2, Smad3, and Smad4), MELK inhibits EMT and increases tumor cell motility, invasion, and metastasis (Cheng et al. 2017). When DNA damage or unreplicated DNA is detected, the cell cycle can be arrested through checkpoint‐mediated mechanisms, and CHEK1 plays a crucial role in this process. NSCLC stem cells rapidly activate CHEK1 to repair damage induced by cisplatin, paclitaxel, or gemcitabine (Bartucci et al. 2012). The AURKA protein is a cell cycle controlled kinase that influences tumor development and progression through its important function in microtubule production and spindle pole stabilization during chromosomal segregation (Du et al. 2021). AURKA's novel substrate, LKB1, when phosphorylated at Ser299, leads to LKB1 and AMPK dissociation, impairing the AMPK signaling pathway and promoting NSCLC growth and migration (Zheng et al. 2018). Despite PLK1's strong expression during mitosis and in many distinct forms of cancer, lower levels of PLK1 in cancer cells disturb cell proliferation and death (Combes et al. 2017). TOP2A increases NSCLC Wnt3a and PD‐L1 expression, which could lead to new immunotherapies and vasculogenic mimicry. Tumor biology is influenced by TOP2A's roles in cell cycle control and apoptosis (Wu, Zhang, et al. 2023). A poor prognosis is associated with KIF11 in NSCLC patients; KIF11 is critical for mitotic spindle formation (Gao et al. 2024) and maintenance (Schneider et al. 2017). KIF11 is also identified as a key gene in the development of BPDE‐associated lung cancer and a potential target for lung cancer prevention and treatment (Ling et al. 2022).

The MR analysis revealed a causal association between elevated AURAB levels and an increased risk of NSCLC in eight hub genes. The Kaplan–Meier survival study revealed that individuals with NSCLC who exhibited high levels of AURKB expression experienced lower OS and PFS. AURKB was found to be significantly overexpressed in NSCLC tissue samples, associated with both shorter PFS and OS in patients (Smith et al. 2005; Yu et al. 2018), aligning with our findings. Of course, it is necessary to point out at this time that the amount of AURKB directly affects the risk of NSCLC, as seen from the MR data. Such a finding may present a new standpoint toward the future treatment of NSCLC.

## 6 Conclusion

‐In summary, this article elucidated the efficacy and potential mechanisms of curcumin in treating NSCLC through comprehensive approaches including systematic review with metaanalysis, network pharmacology analysis, and MR analysis. Focusing on in vivo studies, the meta‐analysis confirmed curcumin's safety and effectiveness, supporting its viability as an NSCLC treatment. By integrating bioinformatics, network pharmacology analysis, and MR, the study detailed the pharmacological mechanisms of curcumin's action. Utilizing the GEO database, the Cybersport algorithm, molecular docking, and MR analysis, it investigated the impact of curcumin on immunity, genes, proteins, and SNPs related to NSCLC. Eight core targets for curcumin were identified and validated across multiple platforms. Notably, MR analysis pinpointed AURKB as both a novel potential therapeutic target for curcumin in NSCLC and a high‐risk factor for the disease. This study provides a foundation for further exploration of curcumin's therapeutic role in NSCLC, highlighting the necessity for future validation through biological experiments and robust evidence‐based medicine.

## Author Contributions

**Yonglu Guo:** conceptualization, data curation, writing – original draft. **Haohao Xu:** data curation, formal analysis, writing – original draft. **Peng Shen:** formal analysis, methodology, writing – review and editing. **Ruijun Cai:** data curation, methodology, project administration, writing – review and editing.

## Disclosure

All claims expressed in this article are solely those of the authors and do not necessarily represent those of their affiliated organizations, or those of the publisher, the editors, and the reviewers. Any product that may be evaluated in this article, or claim that may be made by its manufacturer, is not guaranteed or endorsed by the publisher.

## Conflicts of Interest

The authors declare no conflicts of interest.

## Supporting information


**Data S1:** Supporting Information.

## Data Availability

The original contributions presented in the study are included in the article/Supporting Information, further inquiries can be directed to the corresponding authors.
