# Supplementary material for: Investigating the Potential of Curcumin in the Treatment of Nonsmall Cell Lung Cancer: A Systematic Review With Meta‐Analysis, Network Pharmacology, and Mendelian Randomization
Source: Phytother Res. 2025 Sep 9;39(11):5062–84. doi: 10.1002/ptr.70073 (PMC12605766; doi:10.1002/ptr.70073)
Supplement: Supplementary file 1 — Data S1: Supporting Information. [file PTR-39-5062-s001.zip › Supplementary material.docx]

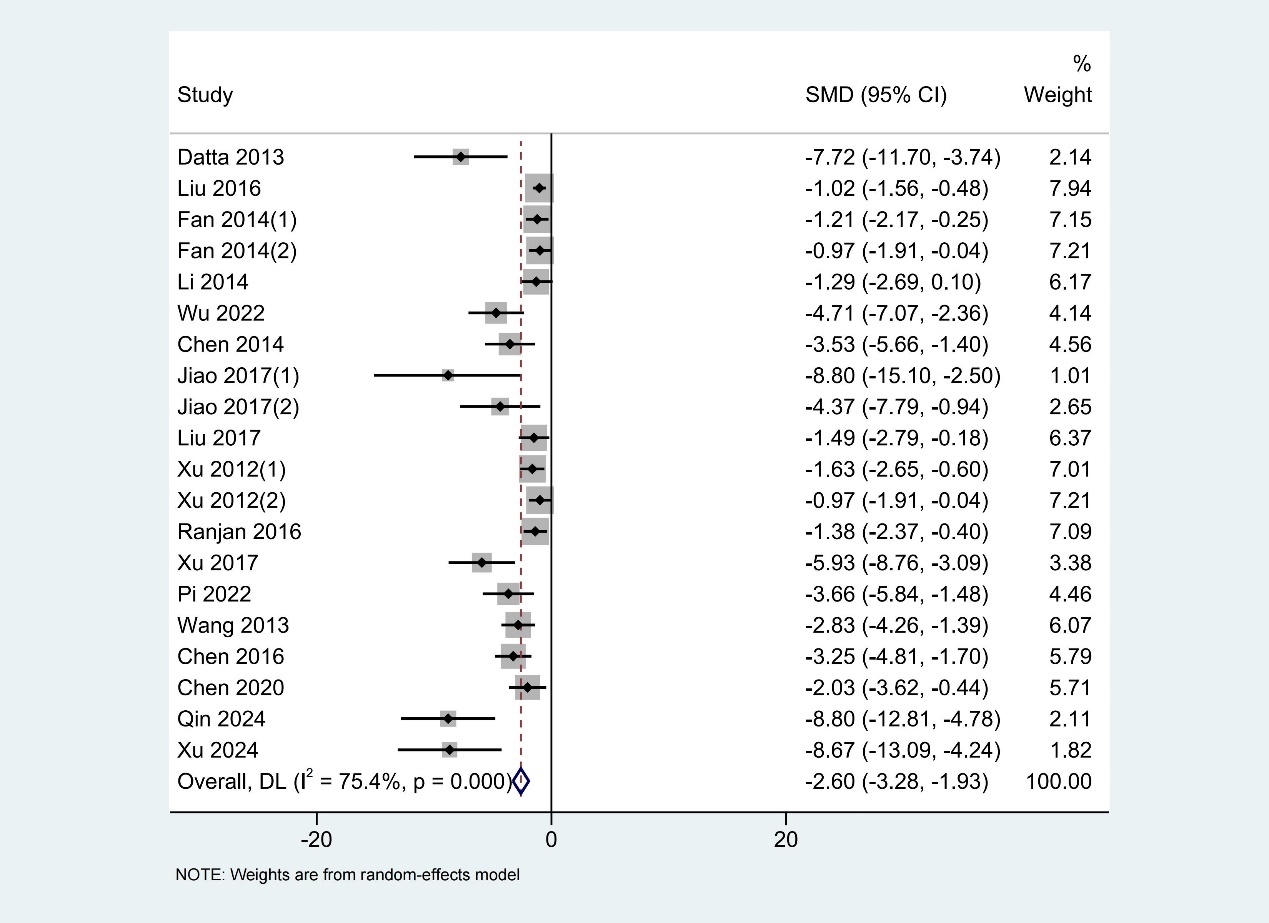


**Supplementary Figure S1** **Forest plot of curcumin effects on** **tumor weight of NSCLC**

Heterogeneity: Tau^2^=1.4189, Chi^2^=77.17; df=19; *p*-value<0.001; I^2^=75.4%. Test for overall effect: Z=-7.563 (*p*-value<0.001)

The findings from the meta-analysis on the impact of curcumin on NSCLC, as presented in **Supplementary Figure S1**, using a random effect model. This analysis specifically examines the outcomes related to tumor weigh, which serves as the secondary outcome of the study. It was observed across 17 included studies, that curcumin significantly reduced tumor weight of NSCLC (*P*< 0.001) (SMD = -2.6; 95% CI: -3.28 to -1.93). These results suggest that all treatments variably inhibited tumor weight growth, indicating curcumin’s potential inhibitory effects on NCSLC. The heterogeneity of the included studies was high (I^2^ = 75.4%).


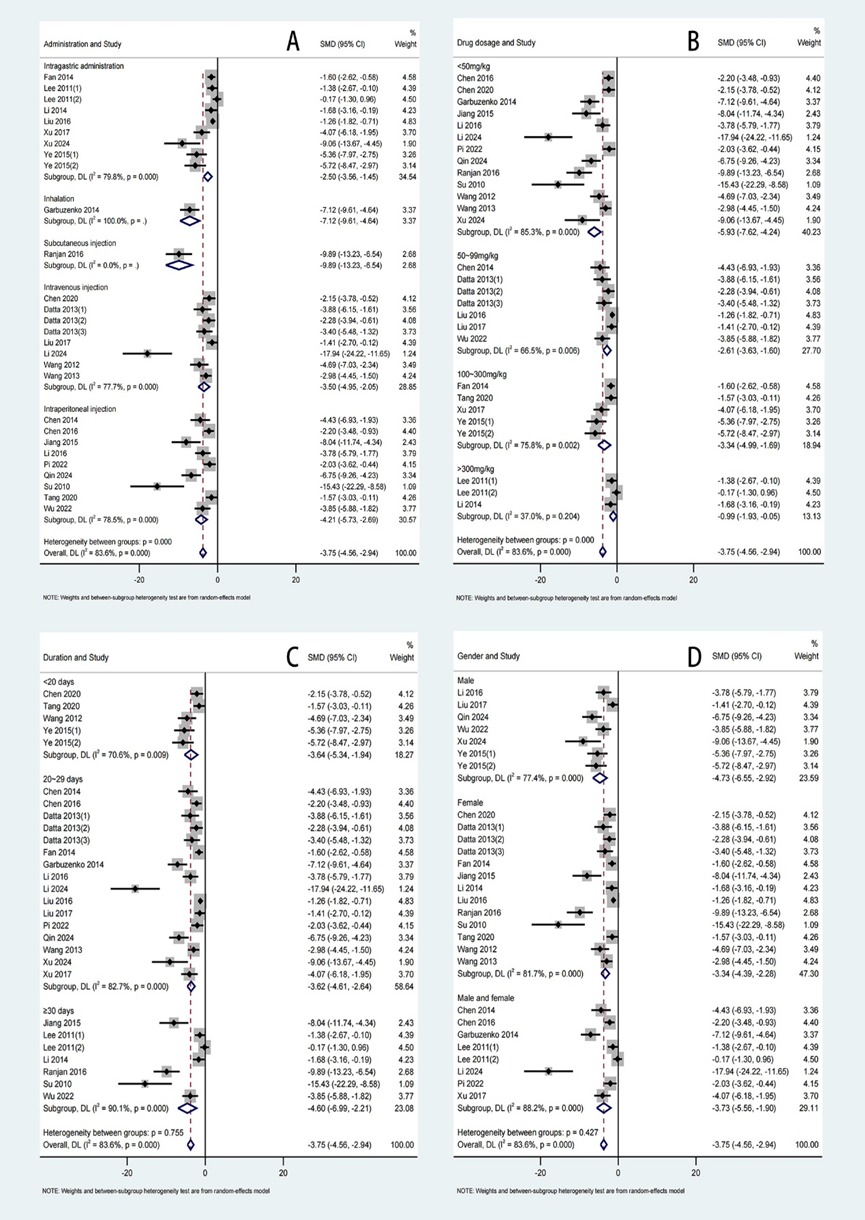


**Supplementary Figure S2** Forest plot of subgroup analysis (with random effect model used for all analyses).

**(A)** Administration method; **(B)** Drug dosage; **(C)** Duration; **(D)** Gender

**1 Administration method**

The administration methods of curcumin given to NSCLC models were also diverse, including intragastric administration (n=7), intraperitoneal injection (n=9), intravenous injection (n=6), subcutaneous injection (n=1), and inhalation administration (n=1), which were all performed with subgroup analysis using a random effect model. Intragastric administration (SMD=-2.5; 95%CI: -3.56 to -1.45; Z =-4.639; *p*-value <0.001), intravenous injection (SMD=-3.5; 95%CI: -4.95 to -2.05; Z=-4.728; *p*-value<0.001) and intraperitoneal injection (SMD=-4.21; 95%CI: -5.73 to -2.69; Z=-5.424; *p*-value<0.001) administration methods all reduced tumor volume growth. All administration method subgroups showed high heterogeneity (**Supplementary Figure S2A** ).

**2 Drug dosage**

The single dose range of curcumin and its formulations is highly variable, ranging from 2.5 to 1000 mg/kg. Based on the combination of single dose and administration method, the study program was categorized into four subgroups (see **Supplementary Figure S2B**), including low dose group <50 mg/kg (n=13), middle dose group 50-99 mg/kg (n=5), higher dose group 100-300 mg/kg (n=4) and high dose group >300 mg/kg (n=2). Subgroup analyses were performed for the four dosing groups using a random effect model, with tumor volume all reduced in low dose group (SMD=-5.93; 95%CI: -7.62 to -4.24; Z=-6.866; *p*-value <0.001), middle dose group (SMD=-2.61; 95%CI:- 3.63 to -1.60; Z=-5.041; *p*-value <0.001), higher dose group (SMD=-3.34; 95%CI: -4.99 to -1.69; Z= -3.965; *p*-value <0.001) and high dose group (SMD=-0.99; 95%CI: -1.93 to -1.69; Z=-2.072; *p*-value=0.038). However, all groups showed low heterogeneity except for the high dose group (I^2^ =37.0%), with I^2^ of the other three groups greater than 50% (**Supplementary Figure S2B**).

**3 Duration**

The treatment course of tumor animal model ranged from 12 to 49 days, the extent of which was great. Therefore, they were divided into 3 groups for subgroup analysis, including less than 20 days (n=4), 20-29 days (n=14), and more than 30 days (n=6). The random effect model analysis showed that tumor volume growth was all reduced in less than 20 days (SMD=-3.64; 95% CI: -5.34 to -1.94; Z=-4.192; *p*-value<0.001), 20-29 days (SMD=-3.62; 95% CI: -4.61 to -2.64; Z=-7.199; *p*-value<0.001) and more than 30 days (SMD=-4.60; 95% CI: -6.99 to - 2.21; Z=-3.767; *p*-value<0.001) group, with I^2^ greater than 50% (**Supplementary Figure S2C**).

**4 Gender**

Among mouse models used for studies, female were only used in 11 studies, male were only used in 6 studies, and female and male were both used in 7 studies (the ratio of female to male selection was not specified in studies). Therefore, they were divided into 3 groups like male (SMD=-4.73; 95%CI: -6.55 to -2.92; Z=-5.112; *p*-value<0.001), female (SMD=-3.34; 95%CI: -4.39 to -2.28; Z = -6.204; *p*-value<0.001) and female and male mixed-sex (SMD=-3.73; 95%CI: -5.56 to -1.90; Z=-3.996; *p*-value<0.001) for subgroup analysis using random effect models. Subgroup analysis of animals in three groups showed a reduction in tumor volume after the experimental intervention, without any gender differences. I^2^ was greater than 50% in all three groups (**Supplementary Figure S2D**).

**
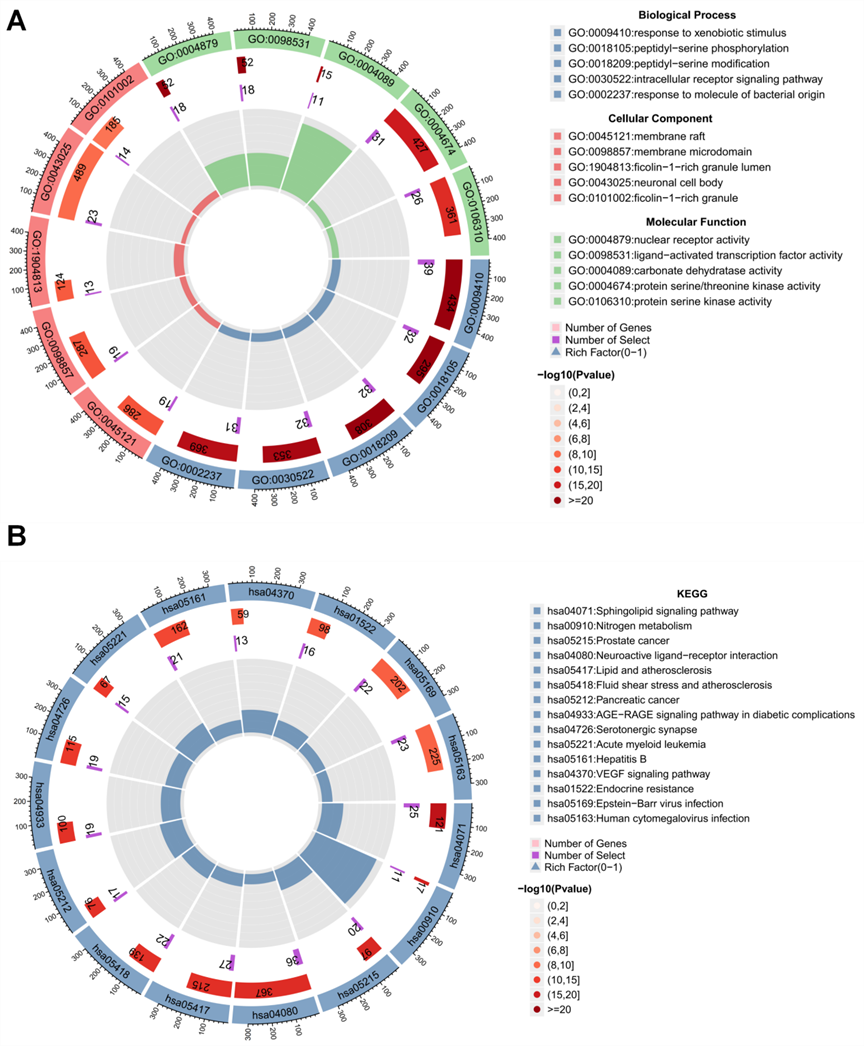
**

**Supplementary Figure S3** Enrichment analysis of curcumin drug targets

The 229 target genes were enriched in Biological processes such as response to xenobiotic stimulus, peptidyl-serine phosphorylation, Cellular Component such as membrane raft, membrane microdomain, Molecular Function such as protein serine kinase activity and protein serine kinase activity (**Supplementary Figure S3A**). KEGG pathway enrichment analysis showed (**Supplementary Figure S3B**) that drug target genes were predominantly enriched for Neuroactive ligand-receptor interaction, Lipid and atherosclerosis (*P* < 0.05).

**(A)** GO enrichment analysis of curcumin drug targets; **(B)** KEGG enrichment analysis of curcumin drug targets


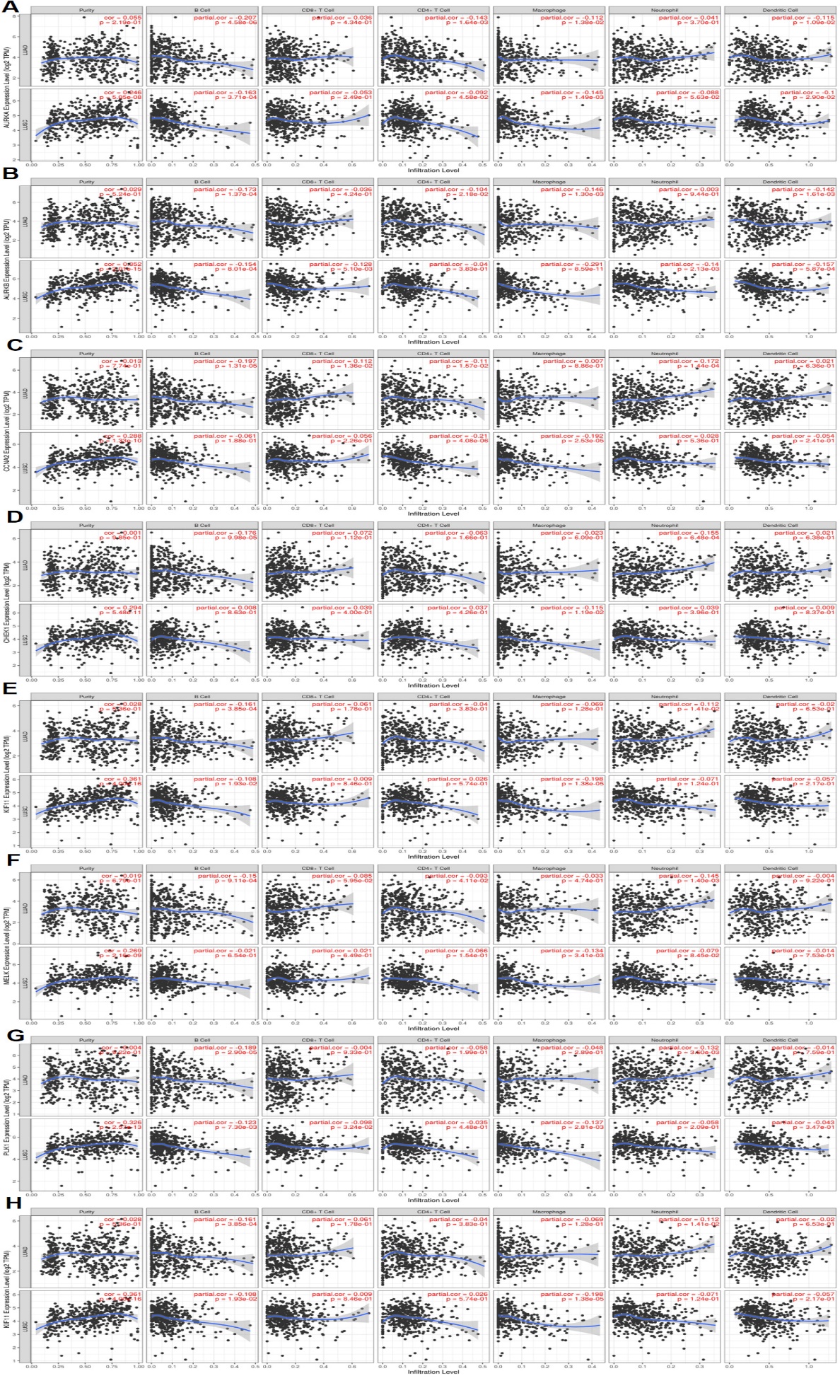


**Supplementary Figure** **S4** Immune infiltration analysis of hub genes

**(A-H)** Correlation between AURKA, AURKB, CCNA2, CHEK1, KIF11, MELK, PLK1, KIF11 and immnue infiltration (*P*＜0.05).

The TIMER website was used to analyze the relationship between 8 hub genes and immune cell infiltration. The results showed that AURKA expression (**Supplementary Figure S4A**) was negatively correlated with the infiltration of B cells, CD4+ T cells, macrophages, and dendritic cells; AURKB expression (**Supplementary Figure S4B**) was negatively correlated with the infiltration of B cells, CD8+ T cells, CD4+ T cells, CD4+ T cells, macrophages and dendritic cells; CCNA2 expression (**Supplementary Figure S4**C) was negatively correlated with the infiltration of B cells and CD4+ T cells, but positively correlated with the infiltration of CD8+ T cells and neutrophils; CHEK1 expression (**Supplementary Figure S4D**) was positively correlated with the infiltration of CD8+ T cells, nephrophils, macrophages and dendritic cells, but negatively correlated with macrophages; KIF11(**Supplementary Figure S4E**) expression was negatively correlated with the infiltration of B cells, macrophages and dendritic cells, but positively correlated with the infiltration of CD8+ T cells; MELK（**Supplementary Figure S4F**） expression was negatively correlated with the infiltration of B cells, CD4+ T cells, macrophages and dendritic cells, but positively correlated with the infiltration of CD8+ T cells; PLK1 (**Supplementary Figure S4G**) expression was negatively correlated with the infiltration of B cells, CD8+ T cells, macrophages and dendritic cells.

**
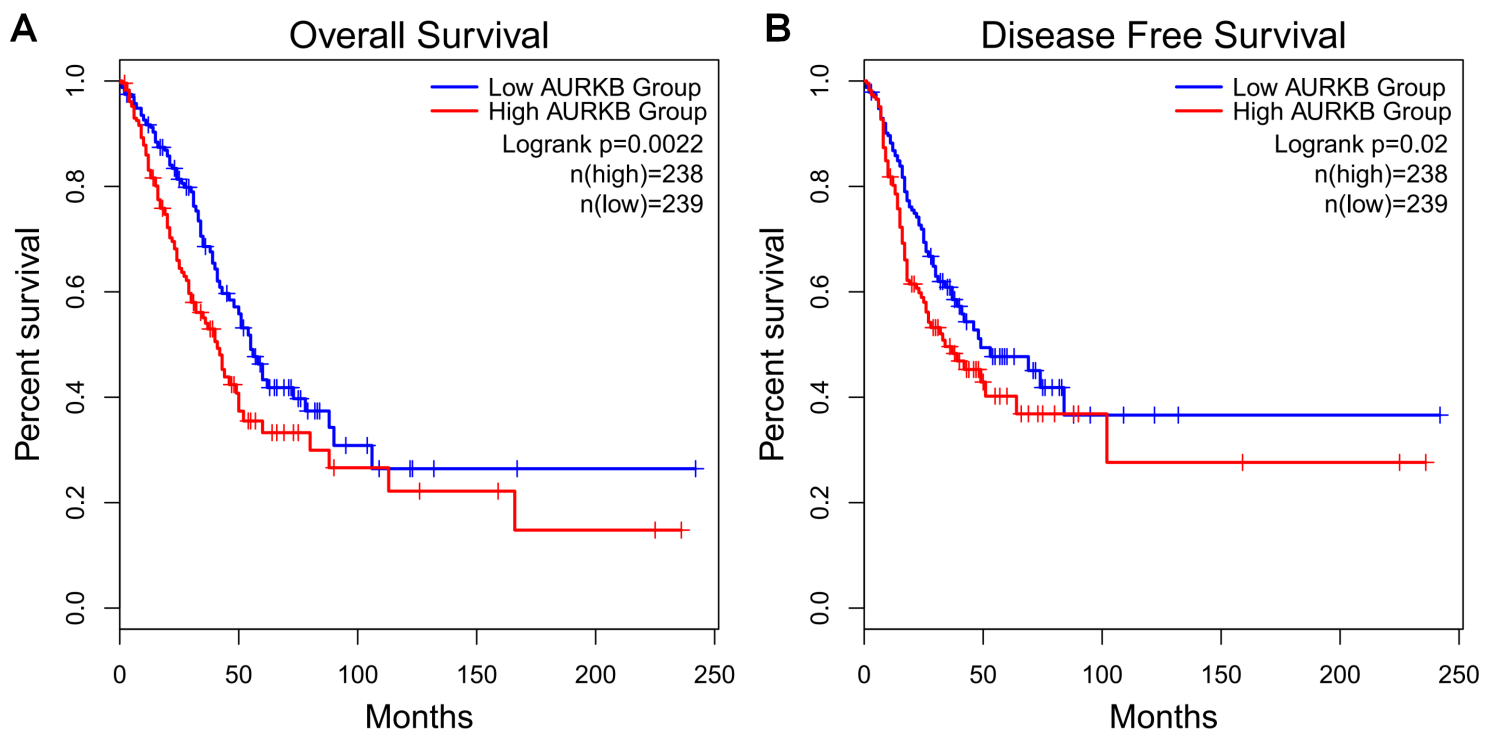
**

**Supplementary Figure S5** Prognostic analysis of AURKB on survival of NSCLC patients

Survival curves showed significantly shorter OS and PFS periods in NSCLC patients with high AURKB expression, indicating that high AURKB expression was associated with poor prognosis in NSCLC patients (*P* < 0.05).

**(A**) Survival analysis curves of the effect of AURKB high and low expression groups on OS in NSCLC patients; **(B)** Survival analysis curves of the effect of AURKB high and low expression groups on PFS in NSCLC patients.

**Supplementary Table S2** **Critical subnetwork details**

| Gene | Degree | MCODE:Clusters | MCODE:Node Status | MCODE:Score |
| --- | --- | --- | --- | --- |
| CCNA2 | 22 | Cluster 1 | Clustered | 7 |
| AURKB | 14 | Cluster 1 | Clustered | 7 |
| MELK | 14 | Cluster 1 | Clustered | 7 |
| CHEK1 | 18 | Cluster 1 | Clustered | 7 |
| AURKA | 14 | Cluster 1 | Clustered | 7 |
| PLK1 | 16 | Cluster 1 | Seed | 7 |
| TOP2A | 18 | Cluster 1 | Clustered | 7 |
| KIF11 | 18 | Cluster 1 | Clustered | 7 |

**Supplementary Table S3 Binding energy of molecular docking**

Unit:kcal/mol

| Molecule name | MOLID | Target | PDBID | Binding energy |
| --- | --- | --- | --- | --- |
| Curcumin | MOL000090 | CCNA2 | 4EOJ | -6.2 |
| Curcumin | MOL000090 | AURKB | 4AF3 | -8.6 |
| Curcumin | MOL000090 | MELK | 5K00 | -9.6 |
| Curcumin | MOL000090 | CHEK1 | 2YEX | -8.4 |
| Curcumin | MOL000090 | AURKA | 6VPM | -7.7 |
| Curcumin | MOL000090 | PLK1 | 4X9R | -7.3 |
| Curcumin | MOL000090 | TOP2A | 5NNE | -6.0 |
| Curcumin | MOL000090 | KIF11 | 3ZCW | -7.9 |

| **Supplementary Table S4 SNPs information for the 8 hub genes** | | | | | | | | | | |
| --- | --- | --- | --- | --- | --- | --- | --- | --- | --- | --- |
| **Gene** | **SNP** | A1 | A2 | **Chr** | **Beta** | **SE** | ***p*** | **eaf** | **pos** | **F value** |
| AURKA | rs6024870 | A | G | 20 | -0.116435 | 0.0219909 | 1.19E-07 | 0.0794099 | 54997568 | 28.03179622 |
|  | rs141294667 | A | G | 20 | -0.257912 | 0.0400758 | 1.23E-10 | 0.0224975 | 54905410 | 41.41373869 |
|  | rs11705555 | C | A | 22 | 0.0791598 | 0.0145199 | 4.99E-08 | 0.213061 | 28206912 | 29.72014226 |
| TOP2A | rs1317082 | G | A | 3 | 0.0742741 | 0.0137723 | 6.94E-08 | 0.247755 | 169497585 | 29.08268052 |
|  | rs184191800 | C | T | 17 | -0.204087 | 0.0393155 | 2.09E-07 | 0.0234221 | 38339135 | 26.94407095 |
| KIF11 | rs113260679 | G | A | 10 | 0.218824 | 0.0289672 | 4.21E-14 | 0.0439801 | 94280911 | 57.06222647 |
|  | rs7092522 | C | T | 10 | 0.100051 | 0.0121612 | 1.92E-16 | 0.607823 | 94342983 | 67.68029264 |
|  | rs17875345 | A | G | 10 | 0.23163 | 0.0287582 | 7.99E-16 | 0.0446275 | 94413667 | 64.86914358 |
|  | rs2249960 | A | G | 10 | 0.0863585 | 0.0171646 | 4.87E-07 | 0.860531 | 94233120 | 25.31125735 |
|  | rs10882098 | T | C | 10 | 0.113136 | 0.0120674 | 6.90E-21 | 0.409159 | 94444793 | 87.89133534 |
|  | rs2007084 | A | G | 15 | -0.109147 | 0.0234482 | 3.24E-06 | 0.0691041 | 90345335 | 21.66575607 |
| CCNA2 | rs75784062 | A | C | 4 | -0.157828 | 0.029169 | 6.27E-08 | 0.043434 | 122757869 | 29.27501311 |
|  | rs970349 | G | A | 4 | -0.0755149 | 0.0142172 | 1.09E-07 | 0.774043 | 122871632 | 28.21043237 |
|  | rs13104823 | C | T | 4 | -0.106426 | 0.0143096 | 1.03E-13 | 0.22129 | 122878536 | 55.31116278 |
|  | rs3762840 | T | A | 4 | -0.143884 | 0.0122902 | 1.17E-31 | 0.366802 | 122746596 | 137.0502046 |
| CHEK1 | rs521102 | A | G | 11 | -0.0601279 | 0.0119053 | 4.41E-07 | 0.522982 | 125514573 | 25.50609478 |
|  | rs484670 | G | A | 11 | -0.0561978 | 0.0124995 | 6.92E-06 | 0.346387 | 125646286 | 20.21277455 |
|  | rs77005011 | C | T | 11 | 0.208138 | 0.0254562 | 2.93E-16 | 0.0577401 | 125710682 | 66.84754267 |
|  | rs76045215 | G | C | 11 | 0.221645 | 0.0248016 | 4.01E-19 | 0.0609825 | 125495746 | 79.85995514 |
|  | rs74573899 | G | C | 11 | 0.207461 | 0.0252841 | 2.30E-16 | 0.0585793 | 125597184 | 67.3209786 |
|  | rs11220091 | T | C | 11 | 0.123647 | 0.0245683 | 4.83E-07 | 0.0624858 | 125361138 | 25.32733177 |
| MELK | rs3819299 | G | T | 6 | -0.0955155 | 0.0209717 | 5.25E-06 | 0.0882038 | 31322367 | 20.74190338 |
| PLK1 | rs72777910 | T | G | 16 | 0.19291 | 0.0344705 | 2.19E-08 | 0.030688 | 23845860 | 31.3168669 |
|  | rs145803744 | T | C | 16 | 0.212596 | 0.0448988 | 2.19E-06 | 0.0178632 | 23811180 | 22.41156831 |
|  | rs73550213 | T | C | 16 | 0.159485 | 0.0339799 | 2.69E-06 | 0.0316321 | 23720652 | 22.02745112 |
|  | rs141277250 | T | C | 16 | 0.215246 | 0.0468108 | 4.27E-06 | 0.0164109 | 23655870 | 21.14134899 |
|  | rs117469707 | T | C | 16 | 0.224406 | 0.045905 | 1.02E-06 | 0.0170732 | 23466646 | 23.89235633 |
|  | rs4967957 | C | T | 16 | -0.173024 | 0.0391456 | 9.86E-06 | 0.0236436 | 23505992 | 19.53503077 |
|  | rs12926135 | A | C | 16 | -0.0933504 | 0.012234 | 2.34E-14 | 0.380426 | 23634750 | 58.21818883 |
|  | rs8049250 | G | C | 16 | -0.350091 | 0.0440017 | 1.77E-15 | 0.0185585 | 23655054 | 63.29795716 |
|  | rs72776163 | G | T | 16 | 0.27956 | 0.0508106 | 3.76E-08 | 0.0138843 | 23728874 | 30.26776699 |
|  | rs30018 | G | A | 16 | 0.170137 | 0.0149495 | 5.21E-30 | 0.195141 | 23408591 | 129.5138243 |
|  | rs72776134 | G | A | 16 | 0.28587 | 0.0444948 | 1.32E-10 | 0.0181705 | 23608516 | 41.27436864 |
|  | rs36047689 | A | G | 16 | -0.0558896 | 0.0119051 | 2.67E-06 | 0.478633 | 23717833 | 22.0377347 |
|  | rs72776116 | C | T | 16 | 0.370357 | 0.0464118 | 1.47E-15 | 0.0166483 | 23534365 | 63.66530515 |
|  | rs6497671 | C | T | 16 | -0.192086 | 0.0159263 | 1.70E-33 | 0.834318 | 23536684 | 145.4565256 |
|  | rs35586 | G | A | 16 | 0.290065 | 0.0140427 | 8.66E-95 | 0.22498 | 23677006 | 426.6397764 |
| AURKB | rs75922939 | A | C | 17 | 0.115129 | 0.0246339 | 2.96E-06 | 0.0621462 | 8156541 | 21.83802685 |
|  | rs150875274 | T | G | 17 | -0.171677 | 0.0323784 | 1.14E-07 | 0.034943 | 8254182 | 28.10921738 |
|  | rs12603646 | C | A | 17 | -0.240206 | 0.0129267 | 4.48E-77 | 0.292457 | 8138643 | 345.2482297 |
|  | rs62063060 | G | A | 17 | -0.233167 | 0.0396011 | 3.91E-09 | 0.0230645 | 8016410 | 34.66056223 |
|  | rs141631668 | A | G | 17 | 0.317445 | 0.040565 | 5.06E-15 | 0.0219144 | 8094710 | 61.22718348 |
|  | rs55844333 | T | C | 17 | -0.213261 | 0.046538 | 4.59E-06 | 0.0166074 | 8047831 | 20.99555472 |
|  | rs12946837 | A | G | 17 | 0.117082 | 0.0251481 | 3.23E-06 | 0.0594615 | 8151178 | 21.67102195 |
|  | rs869773 | T | C | 17 | -0.160298 | 0.0281763 | 1.28E-08 | 0.0466976 | 8192922 | 32.36031347 |
|  | rs1561 | T | C | 17 | -0.177479 | 0.0245839 | 5.22E-13 | 0.0622746 | 8263334 | 52.10948996 |
|  | rs139067265 | G | A | 17 | -0.173829 | 0.0255767 | 1.07E-11 | 0.0572513 | 8069239 | 46.18427904 |
|  | rs11658707 | T | C | 17 | -0.0777641 | 0.0118858 | 6.05E-11 | 0.496293 | 8010719 | 42.79801359 |
|  | rs7219636 | A | G | 17 | -0.0863123 | 0.0160414 | 7.42E-08 | 0.835593 | 8121669 | 28.94639826 |
|  | rs7221194 | G | T | 17 | -0.101114 | 0.0124623 | 4.92E-16 | 0.651575 | 8240800 | 65.82111355 |
|  | rs62063070 | G | C | 17 | -0.141078 | 0.0120467 | 1.12E-31 | 0.40913 | 8034628 | 137.1260662 |

**Supplementary Table S5** Heterogeneity and pleiotropy of MR analysis results

|  | Heterogeneity | | | | Pleiotropy | |
| --- | --- | --- | --- | --- | --- | --- |
| Gene | MR Egger | | IVW | | MR Egger | |
|  | Cochran’sQ | *P*-value | Cochran’sQ | *P*-value | Egger intercept | *P*-value |
| AURKA | 1.28934016 | 0.256169586 | 1.755554839 | 0.415705825 | -0.055316964 | 0.655338415 |
| TOP2A | NA | NA | 0.021792612 | 0.882640138 | NA | NA |
| KIF11 | 0.161845679 | 0.996897142 | 2.785952749 | 0.732944197 | -0.093767992 | 0.180566151 |
| CCNA2 | 0.429784679 | 0.512094622 | 0.469941028 | 0.790594161 | -0.023743336 | 0.874095069 |
| CHEK1 | 1.42443672 | 0.839936871 | 2.074261775 | 0.838777043 | -0.032211599 | 0.465369752 |
| PLK1 | 13.40834209 | 0.416787695 | 13.81032019 | 0.463935991 | -0.021774468 | 0.543239139 |
| AURKB | 8.384705667 | 0.67848406 | 10.20247802 | 0.598202519 | -0.051126543 | 0.204685324 |
